# The Hippo signalling pathway regulates the timing of epithelial cell replacement during *Drosophila* abdominal morphogenesis

**DOI:** 10.1242/jcs.264798

**Published:** 2026-09-15

**Authors:** Kyle Lyon, Samuel A. Manning, Richard Burke

**Affiliations:** ^1^ School of Biological Sciences, Monash University, Wellington Rd, Clayton, VIC 3800, Australia; ^2^ Department of Anatomy and Developmental Biology, Monash University, Wellington Rd, Clayton, Melbourne, VIC 3800, Australia; ^3^ Peter MacCallum Cancer Centre, 305 Grattan St, Melbourne, VIC 3000, Australia

**Keywords:** *Drosophila*, Abdomen, Larval epidermal cells, Apoptosis, Hippo signalling, Morphogenesis

## Abstract

Developmental apoptosis plays an important role in shaping the organs of developing animals, such as occurs during the development of the adult fly abdomen, in which the apoptosis of larval epidermal cells (LECs) is delicately balanced with the proliferation of the presumptive adult epithelial cells. The signalling pathways involved in the coordinated regulation of these processes are at present poorly understood. Here, we demonstrate a role for the Hippo signalling pathway in regulating the timing of LEC clearance during abdominal development. Downregulating Hippo signalling promotes LEC survival into adulthood, whereas upregulating Hippo signalling induces premature LEC removal from the epithelium. Crucially, this effect was found to be dependent upon Hippo pathway-mediated transcription, with increases in the expression of pro-survival genes and increased nuclear localization of the Hippo effector Yorkie occurring in LECs during early pupal development. These findings demonstrate a role for Hippo signalling in abdominal morphogenesis and highlight a biologically relevant example of the function of this pathway as a regulator of developmental apoptosis.

## Teaser

**Summary:** Hippo signalling regulates the timing of larval epidermal cell apoptosis and clearance during *Drosophila* abdominal development through temporal changes in target gene activity, in contrast to the role of this pathway in tissue growth regulation.

## INTRODUCTION

The formation of organs in developing animals requires a delicate interplay between cell proliferation and death. An example of this can be seen in the limbs of many vertebrates, in which the digits of the limb are demarcated by the coordinated clearance of interdigital cells (Zuzarte-Luis and Hurle, 2002). Having multiple, distinct life stages, *Drosophila melanogaster* has proven to be an excellent model organism for studying developmental apoptosis. During the transition from the larval to the adult stage via pupariation, entire larval tissues are destroyed to make way for their adult counterparts. This process can be easily viewed *in vivo* during the morphogenesis of the adult abdomen. While most structures of the adult body are formed from epithelial tissues known as imaginal discs, which are pre-patterned during the larval stage, the adult abdomen is created almost entirely *de novo* during pupal development. This is achieved by the proliferation of the diploid histoblast cells to form the adult epithelium, coupled with the death and removal of polyploid larval epidermal cells (LECs) (Roseland and Schneiderman, 1979). Set aside into clusters of cells known as nests during embryogenesis, histoblasts are held quiescent throughout the larval stages. During early pupal development, rapid histoblast nest proliferation occurs at ∼14-16 h after puparium formation (APF) followed by expansion at 20 h APF. This results in the forced migration and eventual apoptosis of abdominal LECs, with the adult abdomen being fully formed by 36 h APF (Madhavan and Madhavan, 1980). The removal of LECs during this process is dependent on interactions with both neighbouring LECs and migrating histoblasts, with caspase activation in delaminating LECs preceding their basal extrusion and engulfment by circulating haemocytes (Ninov et al., 2007; Nakajima et al., 2011).

The Hippo signalling pathway is an evolutionarily conserved tumour suppressor pathway first discovered in *Drosophila* (Harvey et al., 2003; Pantalacci et al., 2003; Camargo et al., 2007; Dong et al., 2007) with well-known roles associated with growth and apoptosis. The pathway is composed of a kinase cascade that influences the subcellular distribution of the transcriptional co-activator Yorkie (Yki) (Huang et al., 2005). The kinase cascade involves the phosphorylation of the kinase Warts (Wts) by an upstream kinase Hippo (Hpo). This phosphorylation induces Wts to phosphorylate Yki and promote its inactivation through 14-3-3 protein binding (Ren et al., 2010). When the Hippo pathway is less active, a higher proportion of Yki is able to enter the nucleus and interact with the transcription factor Scalloped (Sd) to drive the expression of genes associated with growth and survival (Wu et al., 2008; Goulev et al., 2008). Multiple upstream regulators of the Hippo pathway have been found to interact with cytoskeletal components, resulting in Hippo signalling being recognized as a sensor of mechanical strain in cells (Dupont et al., 2011; Sansores-Garcia et al., 2011; Schroeder and Halder, 2012; Fletcher et al., 2018). The E-cadherin-associated protein Ajuba (Jub) negatively regulates Wts in a tension-dependent manner by tethering Wts to both adherens junctions and basal spot junctions, rendering the kinase inactive (Thakur et al., 2010; Rauskolb et al., 2014; Gaspar et al., 2015; Kroeger et al., 2024). In contrast, the localization of upstream regulators Expanded (Ex), Merlin (Mer) and Kibra at apical cell–cell junctions has been found to have an activating effect on Hippo signalling (Hamaratoglu et al., 2006; Ling et al., 2010; Genevet et al., 2010). In the case of Kibra, medial actomyosin flows during periods of low junctional tension have also been recently reported to promote the Kibra-mediated activation of the Hippo pathway (Tokamov et al., 2023).

Recent work has indicated that changes in mechanical tension are important for the basal extrusion of dead or dying LECs during pupal development. During the latter stages of abdominal morphogenesis, forces enacted by migrating histoblasts compact LECs and drive their removal from the epithelium (Prat-Rojo et al., 2020). Junctional tension has been found to increase in LECs as development progresses, with a reduction of E-cadherin levels and heightened junctional turnover also occurring in the lead-up to cellular extrusion of dorsal midline LECs (Michel and Dahmann, 2020; Teng et al., 2017). Such reductions in junctional E-cadherin levels have previously been found to promote caspase activation in LECs (Hoshika et al., 2020). Additionally, extruding LECs remodel their actin cytoskeleton by increasing medially localized actomyosin to facilitate pulsatile apical contractions (Pulido Companys et al., 2020). How these changes in cytoskeletal tension and architecture are actually translated into apoptotic signals by LECs has, however, not yet been identified. Given the aforementioned role of Hippo signalling as both a regulator of apoptosis and an effector of mechanical strain, we speculated that the Hippo pathway may be involved in this process.

In this study, we detail a role for Hippo signalling in regulating LEC death during abdominal morphogenesis. Manipulating the expression of Hippo pathway components in LECs impacts the timing of their clearance during abdominal development. Importantly, this effect appears to be dependent upon temporal changes in Yki localization and the transcription of pro-survival genes.

## RESULTS

### Altered Hippo pathway activity disrupts abdominal development

**Fig. 1.**
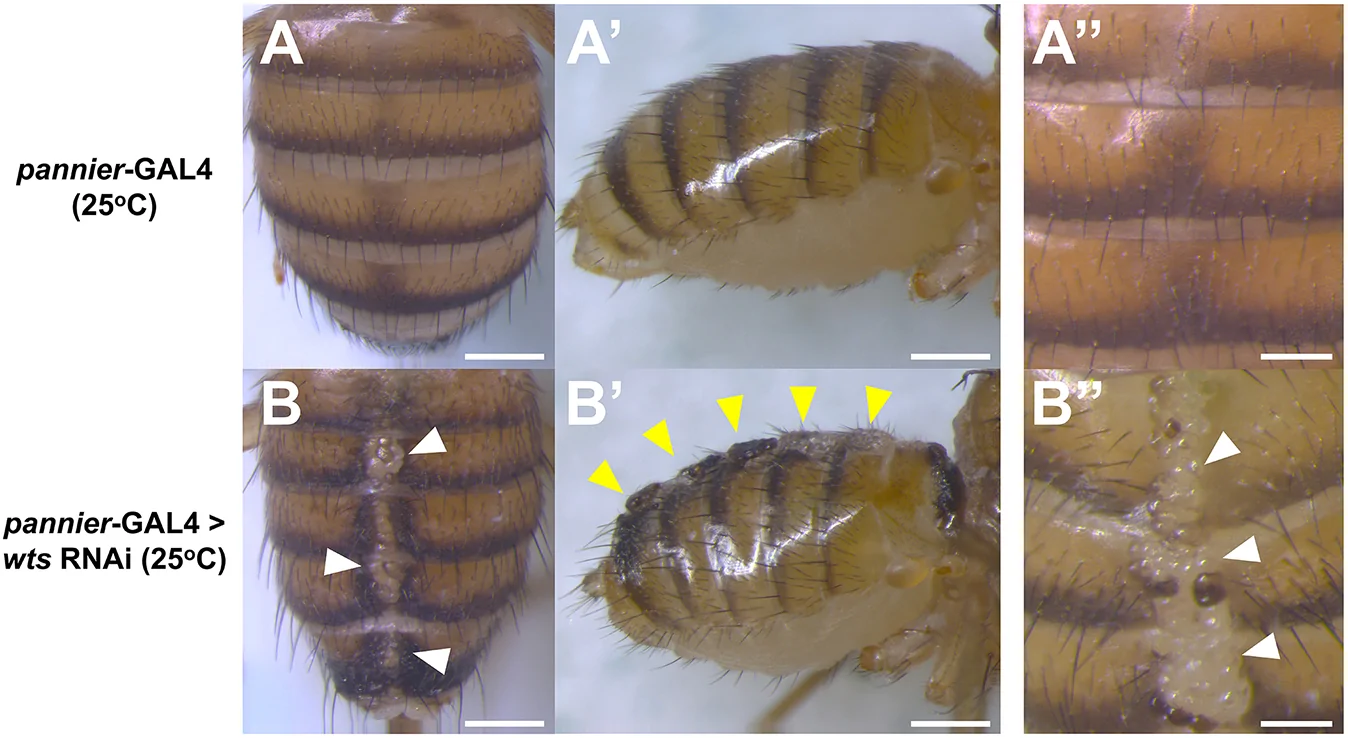
**Downregulated Hippo signalling disrupts the development of the adult abdomen.** (A-B′) Dorsal (A,B) and lateral (A′,B′) views of control (A) and *wts* RNAi (B) adult fly abdomen under *pannier*-GAL4. (A-A″) Control abdomen. (B) Inactivating the Hippo pathway through downregulation of the core kinase *wts* in histoblasts and LECs during both larval and pupal development induces the emergence of circular masses along the length of the dorsal abdomen (white arrowheads). (B′) These masses protrude apically above the adult cuticle (yellow arrowheads). (B″) Higher magnification image of masses (indicated by white arrowheads). Manipulations were made at 25°C. Images for each genotype are representative of five adult females. Scale bars: 250 μm (A-B′); 100 μm (A″,B″).

To first examine whether the Hippo pathway has an effect on LEC clearance during abdominal development, Hippo signalling was downregulated in developing LECs through the expression of an RNA interference (RNAi) line targeting *wts* under control of the *pannier*-GAL4 driver (Fig. 1), which is expressed in LECs in distinct segmental bands along the developing dorsal abdomen (Hoshika et al., 2020). This resulted in splitting of the adult abdominal cuticle, and the protrusion of large circular masses from the underlying epithelium (Fig. 1B,B″, white arrowheads). This phenotype has previously been attributed to tumorigenesis occurring in the abdominal epithelium in response to Hippo pathway downregulation (Mok and Choi, 2022). As *pannier*-GAL4 also drives expression in histoblasts during the later stages of pupal development (Calleja et al., 2000), we sought to investigate this phenotype further to confirm whether it was caused by overgrowth of the adult epithelium or defective LEC apoptosis and clearance.

### Downregulating Hippo signalling prevents LEC clearance and induces LECs to survive into adulthood

**Fig. 2.**
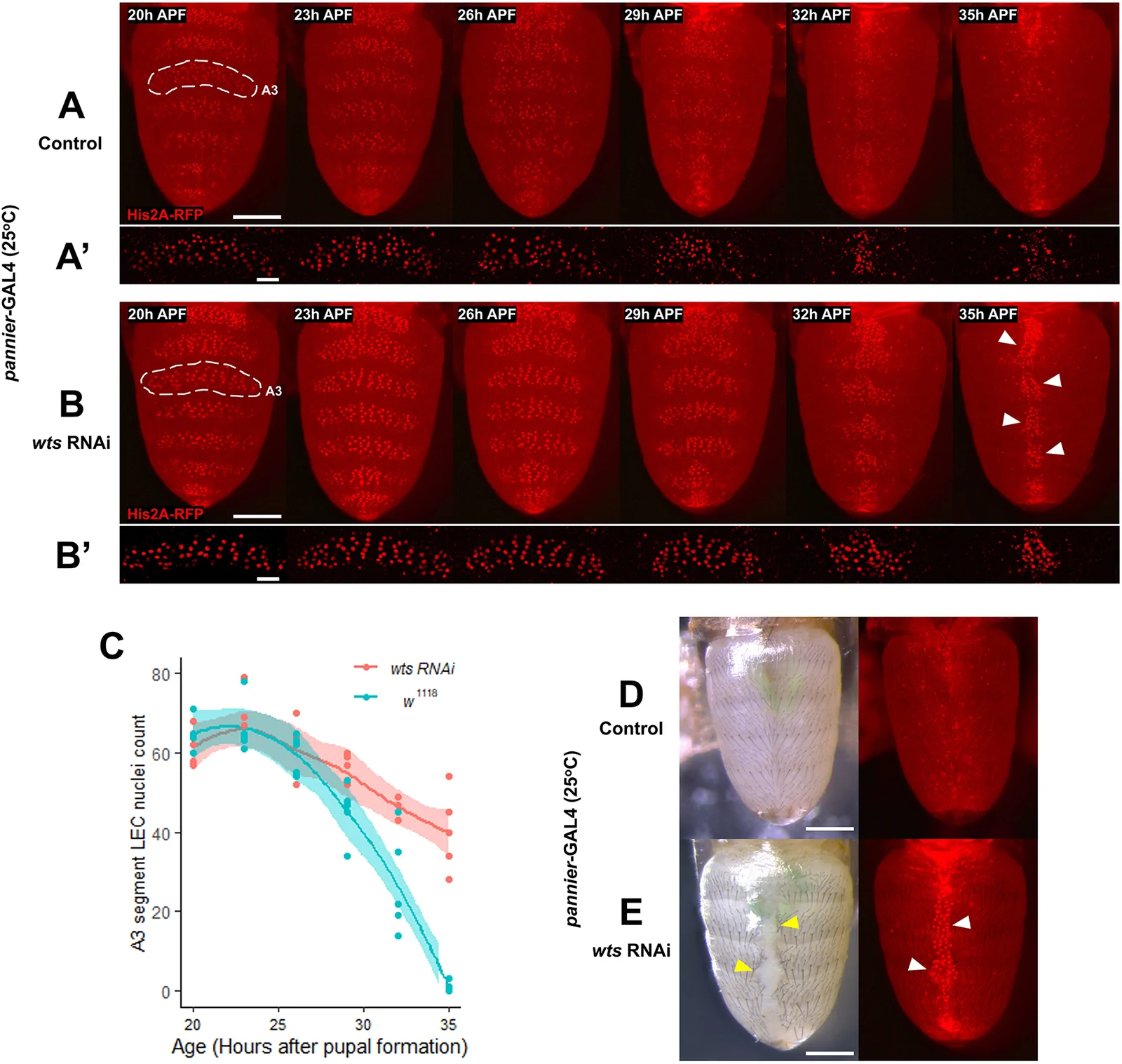
**Reducing Hippo pathway activity in LECs disrupts their clearance during abdominal development.** (A,B) Dorsal view of abdomens of control (A) and *wts* RNAi (B) pupae from 20 to 35 h APF with His2A-RFP highlighting the central stripes of LECs expressing *pannier*-GAL4. Dashed white lines outline the third abdominal (A3) segment in A and B. (A) In a control (*w^1118^*) background, LEC clearance begins from 20 h APF, with nearly all LECs being removed from the epithelium by 35 h APF. (B) Downregulating *wts* in LECs with *pannier*-GAL4 allows their continued persistence throughout development, with LEC nuclei still being clearly visible in the abdomen of *wts* RNAi animals at 35 h APF (white arrowheads). (A′,B′) Higher magnification images of A3 segments in A and B. Background subtraction has been performed to enhance visibility of LEC nuclei. (C) Time course of LEC nuclei counts in the A3 segment of *w^1118^* (blue) and *wts* RNAi (red) pupae. *n*=5 pupae for each genotype at each time point. (D,E) LEC nuclei remain in the fully differentiated abdomen of *wts* RNAi pharate adults (white arrowheads), with these persistent LECs creating a phenotypic defect in the abdomen (yellow arrowheads). Time points indicate age in h APF. Scale bars: 250 μm (A,B,D,E); 100 μm (A′,B′).

To determine whether the abdominal outgrowths caused by *wts* knockdown were occurring due to a disruption in LEC clearance, LEC nuclei were visualized *in vivo* in developing pupae and their fate tracked as development progressed. Focusing on LECs in the third abdominal band (A3) of the developing abdomen, in agreement with previous studies (Madhavan and Madhavan, 1980; Hoshika et al., 2020) it was seen that LEC clearance progresses slowly between 20 and 26 h APF in a control *w^1118^* background, with a rapid decrease in the number of LEC nuclei at later time points preceding their near complete clearance in the A3 segment by ∼35 h APF (Fig. 2A,A′). In comparison, when *wts* knockdown was induced in LECs (Fig. 2B,B′), while the early stages of abdominal morphogenesis were initially similar to that observed in a *w^1118^* background, considerably more LECs were able to survive into the later stages of development (Fig. 2C). This eventually resulted in LEC nuclei being clearly visible in the abdomen at 35 h APF, with masses of LEC nuclei accumulating in clumps along the length of the dorsal midline (Fig. 2B, white arrowheads). These LEC nuclei were still present in pharate adults even after the differentiated adult abdomen had formed (Fig. 2E, white arrowheads). These results indicated that the abdominal phenotype induced by Hippo pathway downregulation occurs due to a disruption in LEC clearance.

**Fig. 3.**
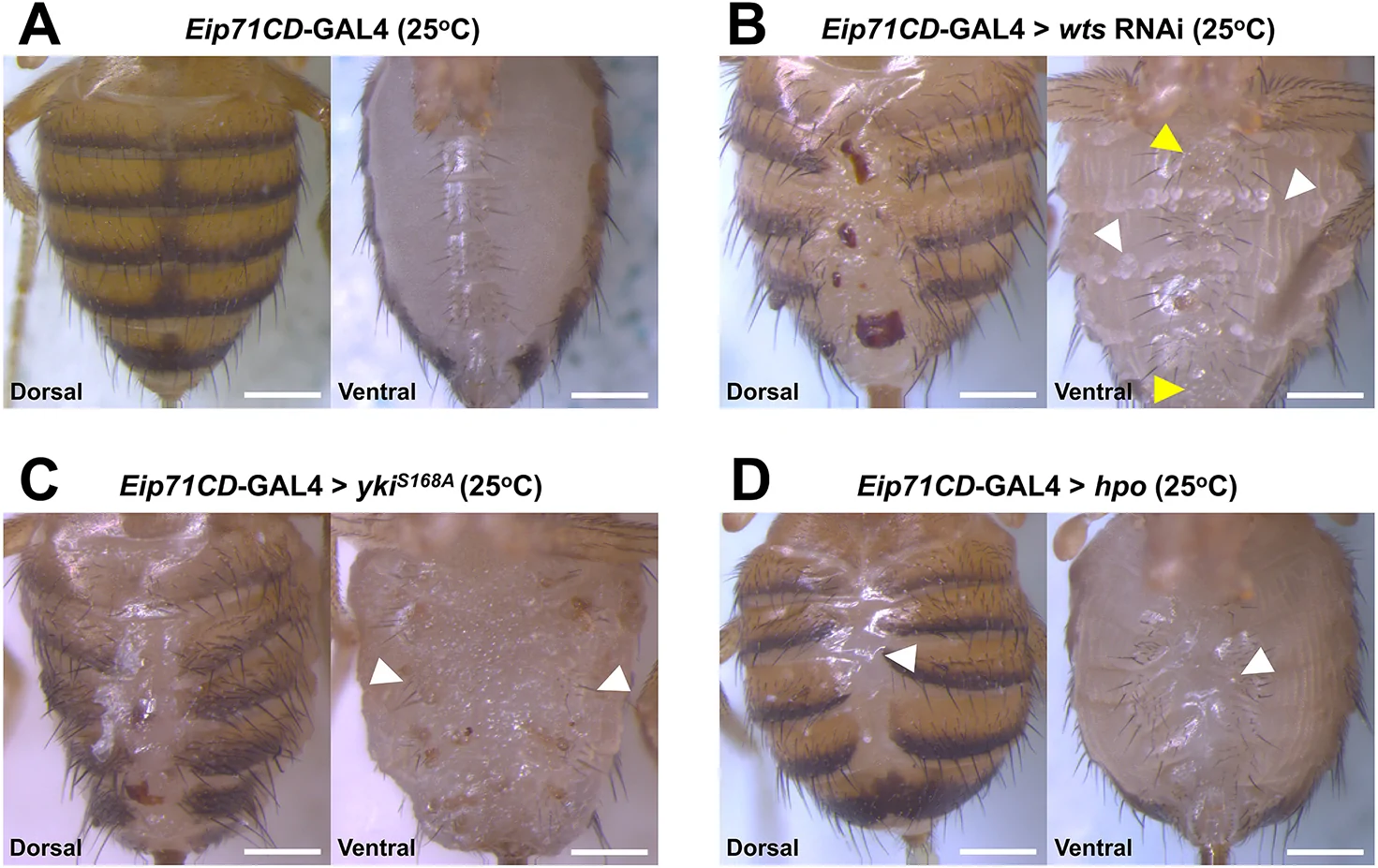
**A LEC-specific GAL4 driver further confirms the effect of disrupting Hippo signalling on LEC clearance.** (A-D) Dorsal and ventral adult abdomens of control (A), *wts* RNAi (B), constitutively active Yki^S168A^ overexpression (C) and *hpo* overexpression (D) animals, all under *Eip71CD*-GAL4 control. Images for each genotype are representative of five adult females. (B) Knocking down *wts* under *Eip71CD*-GAL4 results in LEC persistence occurring along both the dorsal and ventral sides of the adult abdomen. Yellow arrowheads indicate LECs persisting in sternites; white arrowheads indicate LECs in the pleurites. (C) Heavy LEC persistence can be induced in the ventral abdomen through overexpression of a constitutively active form of Yki. Arrowheads indicate sites of displaced sternal bristles. (D) Overexpressing *hpo* induces abdominal clefts in both sides of the adult abdomen (white arrowheads). Scale bars: 250 μm.

The association of the abdominal outgrowth phenotype with LEC persistence was further confirmed using an alternative GAL4 driver. Expression of *pannier*-GAL4 is restricted to distinct segmental bands of LECs on the dorsal side of the abdomen. The *Eip71CD*-GAL4 driver in comparison is expressed in LECs throughout the entire epithelium and does not show expression in histoblasts (Sekyrova et al., 2010), allowing for the effects of disrupting the Hippo pathway to be more broadly and specifically examined in LECs. When *Eip71CD*-GAL4 was used to knockdown *wts* by RNAi, a phenotype characteristic of defective LEC clearance again occurred along the length of the dorsal abdomen, with additional signs of LEC persistence occurring in clumps along the sternites and pleurites of the ventral abdomen (Fig. 3B). While co-expression of a UAS-responsive His2A-RFP confirmed these masses are indeed LECs (Fig. S1B′), the propensity of *Eip71CD*-GAL4 to drive expression in the underlying fat body complicated visualization of the clearance of LECs during pupal development (Fig. S1B). To circumvent this, a transcriptional reporter for the known Hippo target gene *Diap1*, *Diap1*-4.3-GFP, was utilized, as this reporter displays strong expression in LECs with relatively little fat body expression. Using this reporter, it was seen that downregulating *wts* under *Eip71CD*-GAL4 heavily disrupts the progressive clearance of LECs, resulting in LECs persisting into adulthood predominately along the margins at which histoblast nests meet (Fig. S2).

Overexpressing a constitutively active form of Yki, *yki^S168A^*, under *Eip71CD*-GAL4 induced LEC persistence along the dorsal abdomen to a similar extent to that seen from knocking down *wts* (Fig. 3C; Fig. S1C). The ventral abdomen displayed an extreme degree of LEC persistence in the presence of *yki^S168A^* overexpression, as indicated by a prominent displacement of the normally medially localized sternal bristles (Fig. 3C, white arrowheads). Tracking *yki^S168A^* overexpression LEC nuclei with *Diap1*-4.3-GFP further illustrated the extent of this LEC persistence, with ventral histoblast nest expansion appearing to be strongly impeded by persistent LECs (Fig. S3). Lastly, upregulating the Hippo pathway in LECs by overexpression of *hpo* under *Eip71CD*-GAL4 resulted in prominent clefts occurring along both the dorsal and ventral abdomen (Fig. 3D, white arrowheads). Examining LECs in the presence of *hpo* overexpression further revealed that condensed, seemingly apoptotic, nuclei were present in the abdomen of pupae as early as 24 h APF (Fig. S5B), with gaps in the epithelium later becoming apparent between 30 and 35 h APF (Figs S4 and S5D, white arrowheads). This result indicated that premature LEC apoptosis induced by upregulating *hpo* disrupts the normal formation of the adult abdomen. Together, these results demonstrate that LECs are sensitive to changes in Hippo pathway activity during abdominal morphogenesis, with downregulation of Hippo signalling being able to prevent LEC clearance, while upregulation appears to promote it.

### Hippo pathway-dependent transcription is important for LEC survival during pupal development

**Fig. 4.**
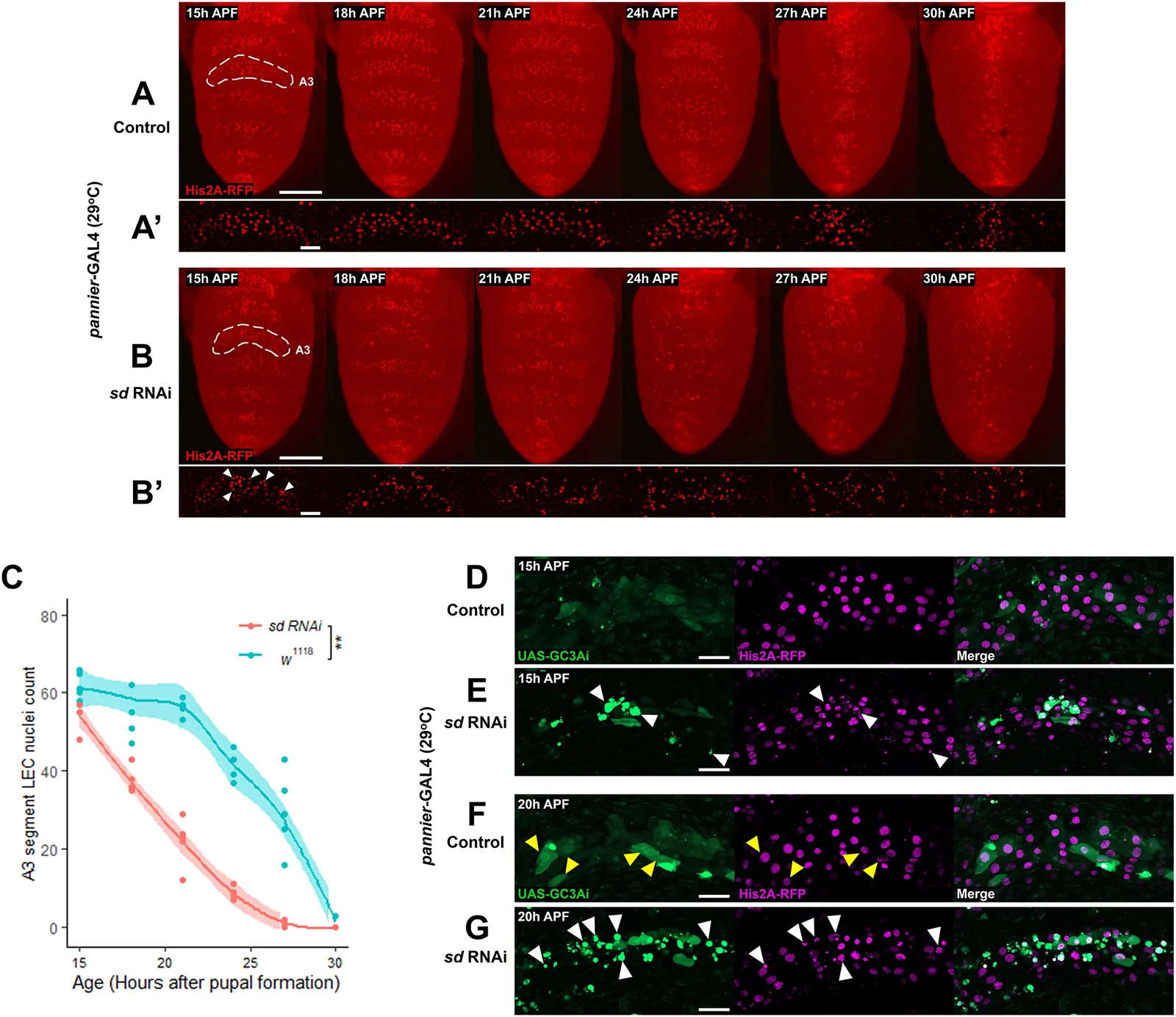
**Downregulating Hippo pathway-dependent transcription in LECs promotes their premature removal from the epithelium.** (A,B) Dorsal view of abdomens of control (A) and *sd* RNAi (B) pupae from 15 to 30 h APF at 29°C, with His2A-RFP highlighting the central stripes of LECs expressing *pannier*-GAL4. Dashed white lines outline the third abdominal (A3) segment in A and B. (A) At 29°C, LEC clearance progressively occurs between 15-30 h APF during normal development. (B) When *sd* is downregulated, the clearance of LECs is accelerated, with fewer LEC nuclei being visible in the abdomen of *sd* RNAi animals as early as 18 h APF. (A′,B′) Higher magnification images of A3 segments in A and B. Background subtraction has been performed to enhance LEC nuclei visibility. White arrowheads in B′ indicate condensed nuclei. (C) Time course of LEC nuclei counts in the third abdominal segment (A3) of *w^1118^* (blue) and *sd* RNAi (red) pupae. Time points indicate age in h APF. *n*=5 pupae for each genotype at each time point. **P<0.01, Kolmogorov–Smirnov test. (D-G) The A3 segment of staged pupae expressing the caspase reporter GC3Ai and a UAS-responsive His2A-RFP under *pannier*-GAL4. (D) Weak GC3Ai fluorescence indicates caspases are mostly inactive at 15 h APF in control LECs. (E) Downregulating *sd* induces premature caspase activation at 15 h APF as indicated by the presence of strong GC3Ai fluorescence (white arrowheads). (F) At 20 h APF, GC3Ai fluorescence is more intense in control LECs (yellow arrowheads). (G) *sd* RNAi LECs at this time point comparatively display mostly dense GC3Ai fluorescent puncta colocalizing with condensed nuclei (white arrowheads). Scale bars: 250 μm (A,B); 100 μm (A′,B′); 50 μm (D-G).

Given the sensitivity of LECs to changes in Hippo pathway activity, we next tested whether Hippo pathway-dependent transcription was playing an active role in abdominal development by examining the effect of knocking down *sd*, the primary transcriptional interaction partner of Yki, in developing LECs. At 25°C, *sd* knockdown had no obvious effects on LEC survival (data not shown). When flies were reared at a higher temperature to maximize RNAi efficiency (29°C), the loss of *sd* expression had a much more noticeable effect on LEC survival. To compensate for the faster developmental rate of flies reared at 29°C, the times at which flies were imaged was shifted forward 5 h to capture a window between 15 and 30 h APF. In control animals, a shift to 29°C had relatively little effect on LEC clearance, aside from the faster rate at which development occurred (Fig. 4A,A′). In contrast, knocking down *sd* in LECs increased the rate at which LECs underwent apoptosis (Fig. 4B), with numerous condensed nuclei being apparent as early as 15 h APF (Fig. 4B′, white arrowheads). This increased rate of apoptosis resulted in LEC clearance occurring significantly earlier, with ∼60% of LECs being removed from the epithelium by 21 h APF compared to ∼10% for control animals (Fig. 4C).

To determine whether these changes in LEC apoptosis are due to an influence of *sd* on caspase activation, the caspase sensor GC3Ai was utilized. GC3Ai is composed of a non-fluorescent GFP protein that refolds to become fluorescent upon cleavage of a DEVD recognition site by effector caspases Dcp1 or Drice, marking cells in which caspase activation has occurred (Schott et al., 2017). Examining GC3Ai at 15 h APF, while only weak GFP fluorescence was visible in a control background (Fig. 4D), multiple cells were strongly GFP positive in the presence of *sd* RNAi (Fig. 4E). Interestingly, while some cells with intact nuclei displayed weak nucleocytoplasmic GC3Ai fluorescence, others with condensed or fragmented nuclei were associated with dense GC3Ai fluorescence overlapping with His2A-RFP fluorescence (Fig. 4E, white arrowheads), indicating that a shift in the cellular distribution of GC3Ai occurs during the latter stages of apoptosis. Following this notion, while at 20 h APF multiple control cells began to display signs of diffuse GC3Ai fluorescence (Fig. 4F, yellow arrowheads), most GC3Ai-positive *sd* RNAi cells presented as dense masses of fluorescence tightly overlapping with condensed and fragmented nuclei (Fig. 4G, white arrowheads). Thus, it appears that Hippo pathway-dependent transcription is actively involved in regulating caspase activation and preventing early LEC apoptosis during abdominal morphogenesis.

### Temporal changes in Hippo-dependent transcription and Yki localization underlie the role of the Hippo pathway in LEC clearance

**Fig. 5.**
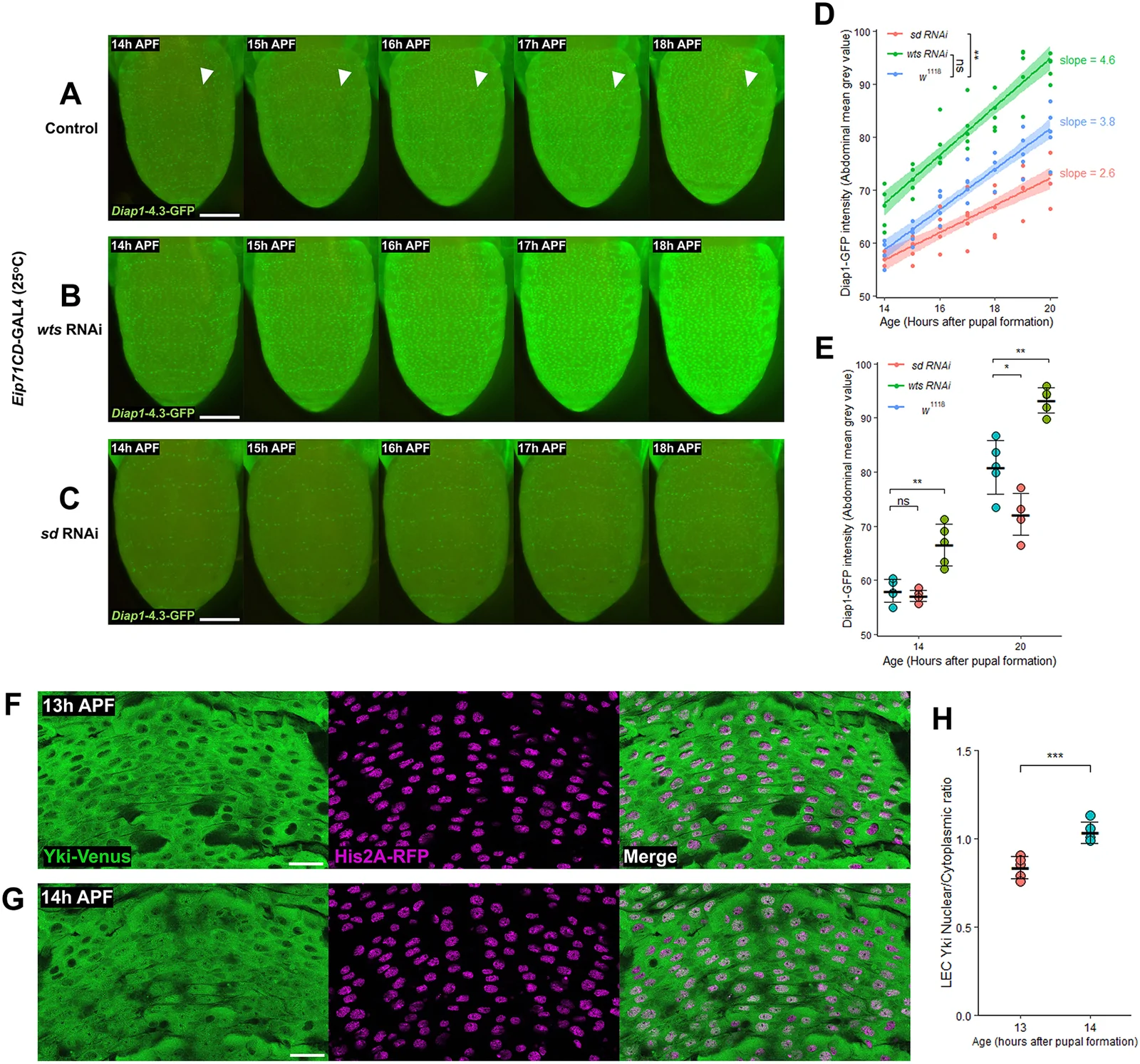
**The expression of *Diap1* scales in LECs between 14 and 20 h APF in a manner dependent upon the activity of the Hippo pathway and Yki localization.** (A-C) Dorsal abdomen of pupae expressing *Eip71CD*-GAL4 in a *w^1118^* (A), *wts* RNAi (B) and *sd* RNAi (C) background from 14 to 18 h APF with *Diap1*-4.3-GFP (green) expression acting as a readout of Hippo pathway transcriptional activity. (A) In the control background, the expression of *Diap1*-4.3-GFP increases progressively between 14 and 18 h APF (*P*<0.0001, linear regression model), with the exception of tendon cells in which *Diap1*-4.3-GFP expression remains relatively constant (white arrowheads). (B) Downregulating *wts* under *Eip71CD*-GAL4 control in LECs results in increased *Diap1*-4.3-GFP expression in LECs. (C) Knocking down *sd* suppresses *Diap1*-4.3-GFP expression in the abdomen. (D) Time course of *Diap1*-4.3-GFP intensity in the abdomen of *wts* RNAi (green), *w^1118^* (blue) and *sd* RNAi (red) pupae. The slope of the linear regression model for each genotype was compared using Welch's *t*-tests. *n*=5 pupae for each genotype at each time point. **P<0.01. (E) Plot depicting initial (14 h APF) and final (20 h APF) *Diap1*-4.3-GFP intensities for each genotype. Comparisons were made using pairwise Student's *t*-tests. *n*=5 pupae for each genotype at each time point. *P<0.05, **P<0.01. (F-H) Endogenous Yki-Venus and His2A-RFP in control early pupal abdominal LECs. (F) Yki-Venus is predominantly localized in the cytoplasm of LECs at 13 h APF. (G,H) In comparison, there is a significant increase in Yki nuclear localization in LECs at 14 h APF. Images depict animals homozygous for Yki-Venus. *n*=5. ***P<0.001, unpaired two-tailed Student's *t*-test. Data are represented as mean±s.d. ns, not significant. Scale bars: 250 μm (A-C); 50 μm (F,G).

While our results indicated that Hippo signalling is directly involved in LEC clearance, the transcriptional targets of the Hippo pathway regulating LEC survival still remained to be identified. One transcriptional target of particular interest was the anti-apoptotic E3 ubiquitin ligase Diap1 (Hay et al., 1995). Diap1 has been demonstrated to influence LEC survival (Kester and Nambu, 2011) and is known to be a canonical target of the Hippo signalling pathway (Zhang et al., 2008). A fluorescent Hippo-dependent transcriptional reporter for *Diap1* expression (*Diap1*-4.3-GFP) indicated that *Diap1* was endogenously expressed in abdominal LECs in a temporally sensitive manner, with GFP intensity found to increase significantly with age during the early stages of abdominal development (adjusted R^2^=0.86, *P*<0.0001, linear regression model) (Fig. 5A,D). This temporal change in *Diap1* expression was restricted to segmental LECs, with the differentiated tendon cells separating each abdominal segment displaying relatively consistent *Diap1* expression across development (Fig. 5A, white arrowheads). In response to *wts* knockdown, while both the initial and final intensities were significantly higher than that seen in a control background (Fig. 5B,E), the rate at which GFP intensity increased was relatively unchanged (Fig. 5D). In comparison, when *sd* was knocked down in LECs the rate at which *Diap1*-4.3-GFP expression increased in the abdomen was significantly reduced (Fig. 5C,D), resulting in significantly lower GFP intensity at 20 h APF (Fig. 5E). While some *Diap1*-4.3-GFP expression was still visible in *sd* knockdown LECs, this residual *Diap1* expression could potentially be attributed to incomplete *sd* knockdown, given the aforementioned ineffectiveness of the RNAi line at 25°C. Tendon cells remained unaffected due to *Eip71CD*-GAL4 not being expressed in this cell type (Fig. S6). Knocking down *sd* at 29°C appeared to enhance the reduction of *Diap1*-4.3-GFP expression seen at 25°C, with large gaps forming in the epithelium at sites of low *Diap1*-4.3-GFP expression during the later stages of pupal development (Fig. S7C,D, white arrowheads) in a manner similar to that seen for *hpo* overexpression (Figs S4 and S5D). Consistent with the induction of these gaps, *sd* RNAi induced cuticular clefts with reduced penetrance at 29°C (Fig. S7E), with this phenotype being recapitulated using two copies of *sd* RNAi at 25°C (Fig. S7F).

As an increase in Hippo pathway-dependent transcription was observed in early pupal LECs, we then examined whether this change in Hippo pathway activity could be reflected in Yki activity. The primary function of the Hippo pathway is to regulate the subcellular localization of Yki, with the phosphorylation of Yki by Wts acting to decrease the nuclear import of Yki and promote its accumulation in the cytoplasm. When viewing an endogenously Venus-tagged version of Yki in LECs immediately after head eversion (∼13 h APF), Yki was localized predominately in the cytoplasm [nuclear/cytoplasmic (N/C) ratio 0.83] (Fig. 5F). At ∼14 h APF, a shift in Yki subcellular localization occurred (Fig. 5G), with segmental LECs displaying significantly more nuclear Yki (N/C ratio 1.03) at this time point (Fig. 5H). The timing of this shift in Yki localization roughly coincides with the observed scaling of *Diap1* expression in LECs. As such, it appears that Yki enters the nucleus in LECs during the early stages of abdominal development, potentially allowing for the Hippo pathway to direct the transcription of pro-survival genes such as *Diap1*.

### Caspase activation in LECs is inhibited when the Hippo pathway is downregulated

**Fig. 6.**
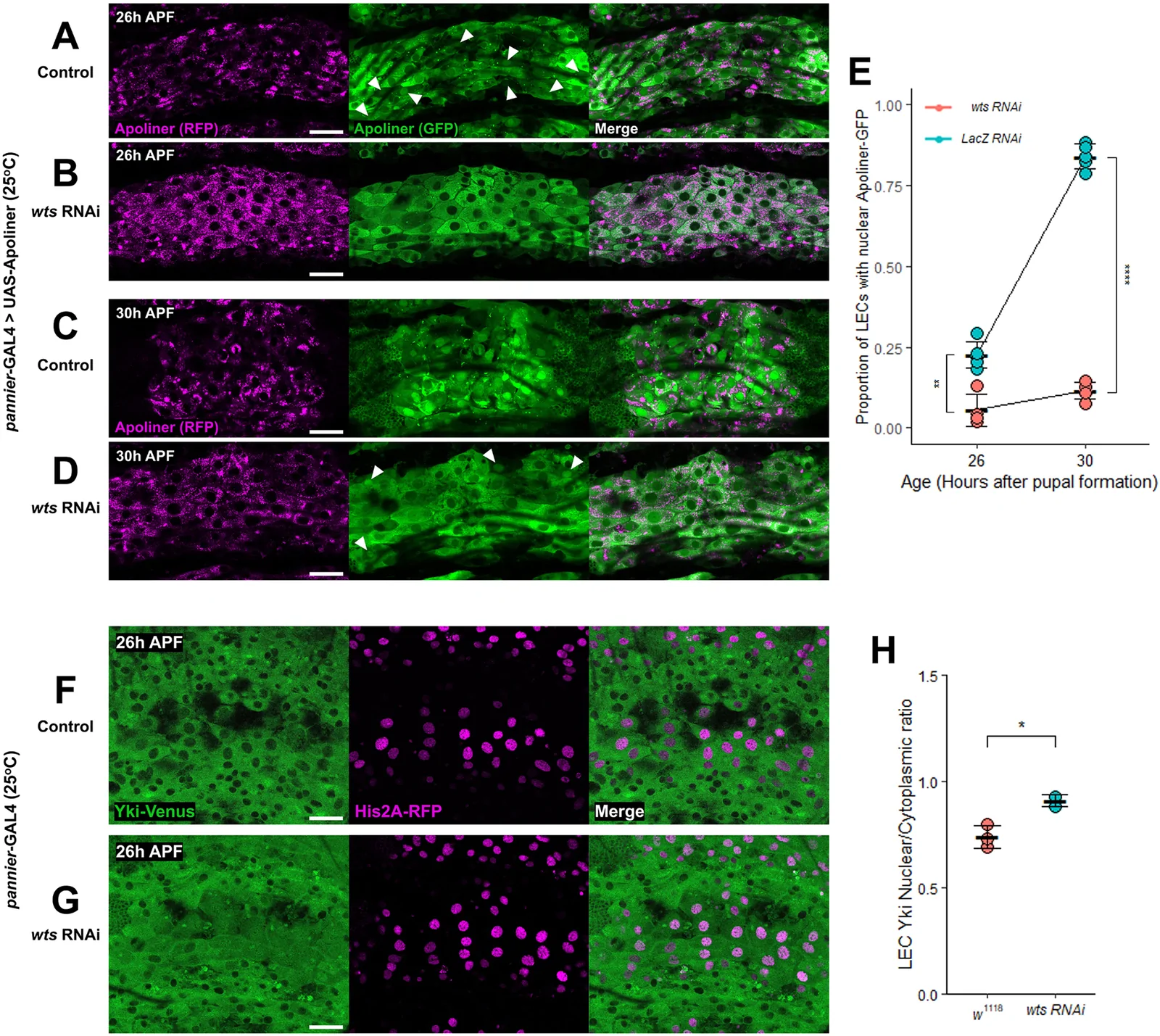
**The activation of caspases in LECs is reduced when the Hippo pathway is downregulated.** (A-G) Expression of the caspase reporter Apoliner or Yki-Venus with UAS-His2A-RFP under *pannier*-GAL4 in staged pupal LECs of control (*lacZ* RNAi in A,C; *w^1118^* in F) and *wts* RNAi (B,D,G) animals at 26 h (A,B,F,G) or 30 h APF (C,D), with quantification shown in E. Cleavage of Apoliner results in the separation of a membrane-targeted RFP and nuclear-targeted GFP, allowing GFP to enter the nucleus. (A) In the control background, many LECs display signs of nuclear GFP at 26 h APF (white arrowheads), indicative of caspase activation occurring in these cells. (B,E) Significantly fewer LECs display signs of nuclear GFP in the presence of *wts* knockdown at this time point. *n*=4. **P<0.01, unpaired two-tailed Student's *t*-test. (C) 84% of control LECs show nuclear Apoliner GFP at 30 h APF. (D,E) In contrast, in the presence of *wts* RNAi at 30 h APF only 11% of LECs present with nuclear Apoliner GFP (white arrowheads). *n*=5. ****P<0.0001, unpaired two-tailed Student's *t*-test. (F) Yki-Venus is predominantly cytoplasmic at 26 h APF. (G) LECs experiencing *wts* knockdown display relatively high degrees of nuclear Yki-Venus compared to their wild-type neighbours. Images depict animals heterozygous for Yki-Venus. (H) *wts* knockdown LECs maintain significantly higher levels of nuclear Yki relative to controls. *n*=3. *P<0.05, unpaired two-tailed Student's *t*-test. Experiments were performed at 25°C. Data are represented as mean±s.d. Scale bars: 50 μm.

Since Diap1 is involved in caspase inhibition, we speculated that LEC persistence in a *wts* knockdown background may be due to an inhibition of caspase activation. To test this notion, the caspase reporter Apoliner was used to view caspase activation in *wts* knockdown LECs. Apoliner is composed of a membrane-targeted RFP and a nuclear-targeted GFP linked together by a caspase-cleavable site, with the separation of these two fluorescent proteins and nuclear localization of GFP being an indicator of caspase activity *in vivo* (Bardet et al., 2008). Apoliner has previously been used to show that a flush of caspase activation occurs in LECs at ∼26 h APF (Michel and Dahmann, 2020). This roughly corresponds to the time point at which LEC clearance diverged from normal under *wts* knockdown (Fig. 2C). At 26 h APF in a control (*lacZ* RNAi) background, caspase activation was consistently observed in LECs in the A3 abdominal segment, with ∼23% of cells on average displaying nuclear GFP (Fig. 6A,E, white arrowheads). In comparison, when *wts* was knocked down in LECs using *pannier*-GAL4, significantly fewer LECs displayed signs of caspase activation at this time point, with nuclear Apoliner GFP being visible in only ∼5% of cells (Fig. 6B,E). This blockage of caspase activation was even more apparent at 30 h APF, a time point in which ∼84% of LECs displayed signs of caspase activation in a control background (Fig. 6C,E). While Apoliner cleavage appeared to occur in a subset of *wts* RNAi LECs at this time point (∼11%) (Fig. 6D, white arrowheads), the extent of this activation remained significantly lower relative to controls (Fig. 6E). The subcellular distribution of Yki-Venus was visualized at 26 h APF to further determine whether differences in caspase activation observed at this time point would be reflected by changes in Yki localization. In a *w^1118^* background, Yki-Venus was predominantly cytoplasmic in LECs at 26 h APF (N/C ratio 0.74) (Fig. 6F,H). Knocking down *wts* in LECs resulted in significantly higher levels of nuclear Yki-Venus (N/C ratio 0.91) (Fig. 6G,H). Thus, it appears that downregulating the Hippo pathway impedes caspase activation in LECs by maintaining nuclear Yki localization.

### Additional Hippo pathway targets may also influence LEC clearance

**Fig. 7.**
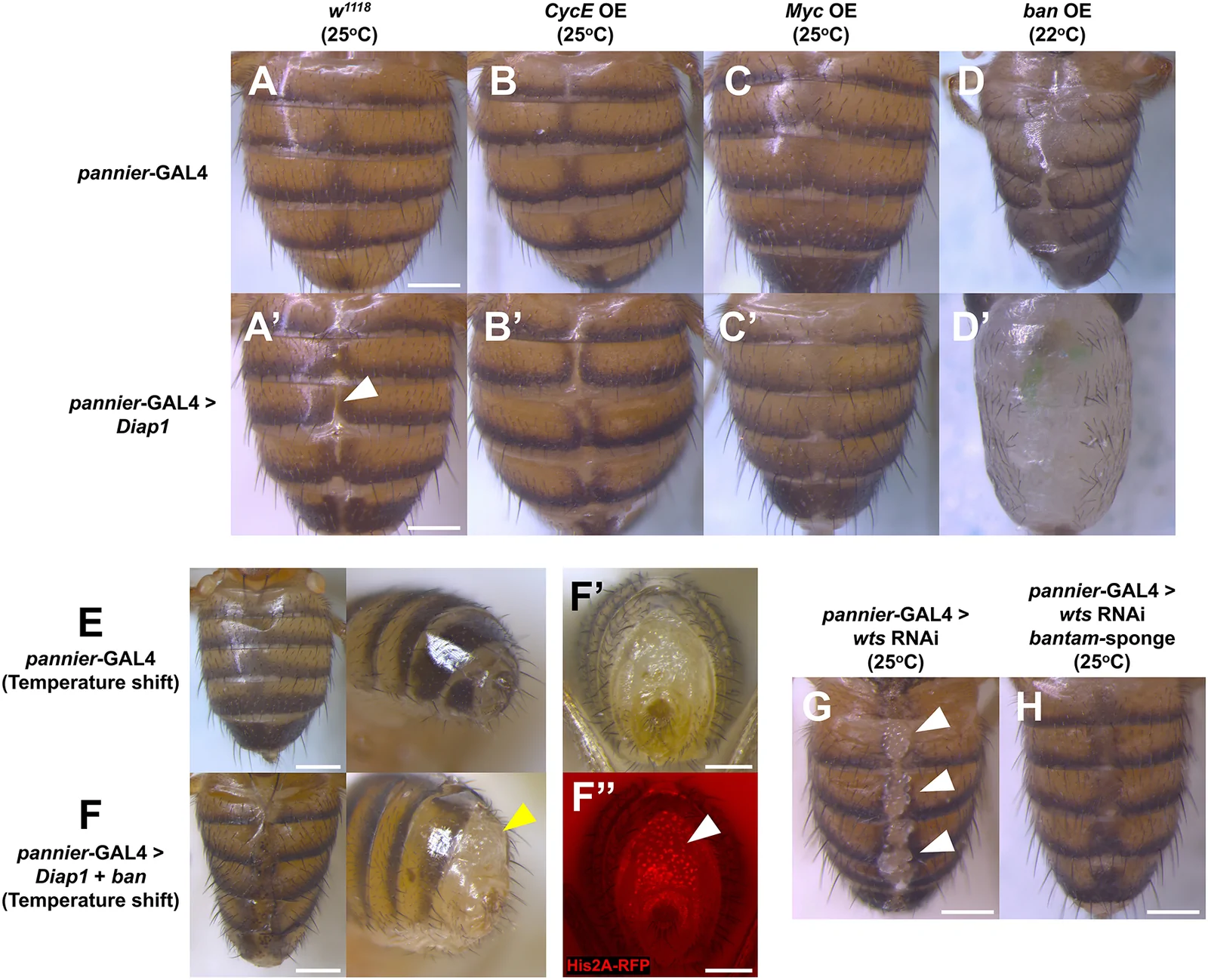
**Combined overexpression of *Diap1* and *ban* strongly disrupts abdominal development.** (A) A *pannier*-GAL4 control abdomen. (A′) Overexpression of *Diap1* induces a cuticular split along the length of the dorsal midline. (B-C′) Overexpression of either *CycE* or *Myc* by themselves or in combination with *Diap1* has relatively little effect on abdominal development. (D) *ban* overexpression disrupts abdominal development, leading to cuticular clefts being seen in the dorsal side of the abdomen. (D′) When *Diap1* and *ban* overexpression are combined, lethality is induced, with pharate adults presenting signs of extreme bristle disruptions along the length of their abdomen. (E) Control abdomen. (F) Adults overexpressing both *Diap1* and *ban* obtained from a temperature-shift experiment displayed large clumps of tissue protruding from the posterior ends of their cuticles (yellow arrowhead). (F′,F″) A UAS-responsive His2A-RFP marks LEC nuclei in these clumps (white arrowhead). (G) At 25°C, *wts* RNAi under *pannier*-GAL4 induces LEC persistence along the midline of the dorsal abdomen. (H) Downregulating *ban* expression using *bantam*-sponge entirely rescues LEC persistence associated with downregulating *wts* under *pannier*-GAL4. Crosses were performed at 22°C for *ban* overexpression due to early pupal lethality at higher temperatures. Temperature shifts involved raising larvae at 18°C before shifting to 29°C for pupal development. Scale bars: 250 μm (A-H,A′-D′); 200 µm (F′,F″).

Although we have found that changes in caspase activation occur in LECs experiencing Hippo pathway downregulation, changes in *Diap1* expression are unlikely to be the sole factor driving the LEC persistence phenotype. While previous research has indicated that blocking apoptosis by overexpressing *Diap1* or p35 enhanced LEC survival (Fig. 7A,A′) (Ninov et al., 2007; Kester and Nambu, 2011), a subset of these surviving LECs have been reported to still undergo basal extrusion, persisting underneath the adult epithelium rather than within it. In contrast, LECs that persist following *wts* knockdown do not appear to experience basal extrusion, given that surviving cells instead form mounds that protrude apically above the adult epithelium (Fig. 1B′, yellow arrowheads). Other Hippo pathway targets in addition to *Diap1* are thus likely to be influencing the manner in which these LECs survive into adulthood. In an attempt to further recapitulate the *wts* knockdown phenotype in LECs, the canonical Hippo pathway targets *Cyclin E* (*CycE*), *Myc* and *bantam* (*ban*) were overexpressed in the dorsal midline using *pannier*-GAL4, both alone and in combination with *Diap1* overexpression. While overexpression of neither *CycE* nor *Myc* induced any effect in the abdomen (Fig. 7B-C′), overexpression of *ban* induced prominent cuticular clefts along the length of the dorsal abdomen at 22°C (Fig. 7D). Combined overexpression of *ban* with *Diap1* resulted in early pupal lethality at all temperatures, but animals were able to develop into pharate adults at lower temperatures. Examining the abdomen of pharate adults overexpressing both *ban* and *Diap1* at 22°C revealed a large gap in bristles occurring along the midline of the dorsal abdomen, potentially indicative of LEC persistence (Fig. 7D′). Through the usage of a temperature-shift experiment, in which *Diap1* and *ban* overexpression were restricted to the pupal stages of development, viable adults could be obtained. Notably, examining the posterior-most end of these flies revealed large clumps of tissues protruding from underneath the adult cuticle (Fig. 7E,F, yellow arrowhead), with the expression of His2A-RFP in these clumps revealing their identity as persistent LECs (Fig. 7F′,F″, white arrowhead).

Downregulating *ban* expression using *bantam*-sponge, a construct encoding a fluorescent dsRed protein with ten recognition sites for the mature *ban* microRNA in its 3′ UTR (Becam et al., 2011), further indicated that altering *ban* expression can influence LEC survival. While the expression of *bantam*-sponge alone under *pannier*-GAL4 had no obvious phenotypic effect at 25°C (Fig. S8B), in the presence of *wts* RNAi the LEC persistence induced by Hippo pathway downregulation (Fig. 7G) was entirely ameliorated (Fig. 7H). Under *pannier*-GAL4, *bantam*-sponge was also able to suppress the abdominal phenotype produced by *Diap1* overexpression (Fig. S8E). Additionally, upregulating *ban* expression under *Eip71CD*-GAL4 strongly exacerbated the LEC persistence associated with *wts* RNAi, with surviving LECs appearing to remain within the epithelium rather than protruding apically (Fig. S8I,I′). A reporter for *ban* expression (*ban*-3-GFP) also revealed that *ban* was endogenously expressed in LECs. Knocking down *sd* under the control of *pannier-GAL4* caused a strong reduction in *ban-3-*GFP expression levels compared to controls, indicating that expression of *ban* in LECs is dependent on Sd-mediated transcription (Fig. S9A,B). In contrast to *Diap1* however, *ban* expression remained relatively consistent in LECs throughout the early stages of pupal development (Fig. S9C,D). From the combination of these phenotypic results, we conclude that *Diap1* and *ban* are two Hippo pathway target genes potentially influencing *wts* knockdown-induced LEC survival.

## DISCUSSION

**Fig. 8.**
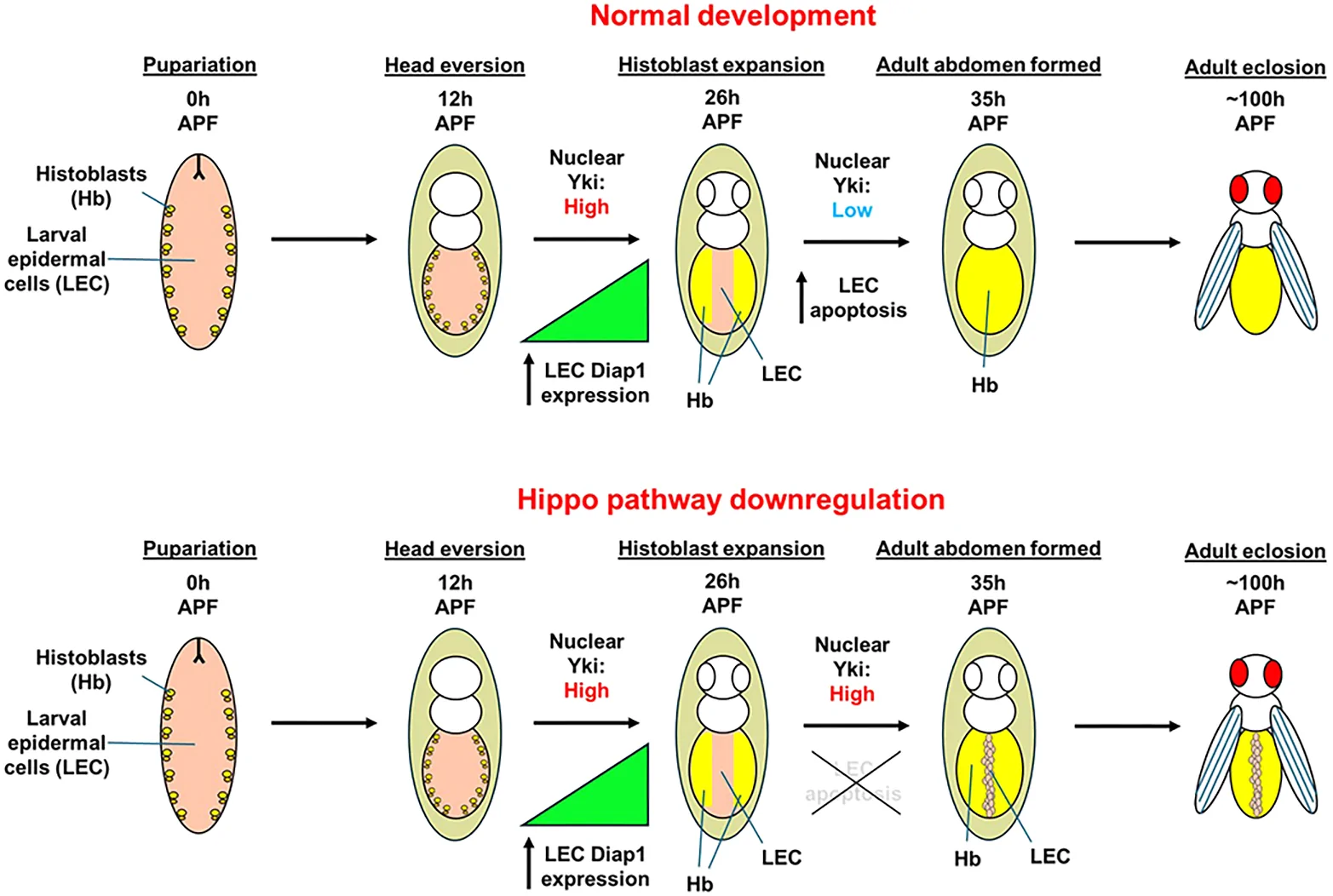
**The Hippo pathway regulates the timing of LEC clearance through temporal changes in Yki localization.** Model depicting the role of Hippo signalling during abdominal development. Under normal conditions, a shift in Yki localization towards the nucleus at ∼14 h APF induces an upregulation of pro-survival genes to prevent early LEC extrusion. Later in development, when the histoblast nests have begun to spread, a shift of Yki back towards the cytoplasm downregulates pro-survival gene expression, allowing LECs to undergo apoptosis and be removed from the epithelium. When components of the Hippo pathway are downregulated, the maintenance of high nuclear Yki levels during late abdominal development prevents LEC apoptosis, causing their persistence into adulthood.

By following LEC clearance throughout pupal development, it is evident that manipulating Hippo signalling can influence the survival of LECs during abdominal morphogenesis. Decreasing the transcription of pro-survival genes in LECs by downregulating *sd* induces early LEC death, whereas downregulating the Hippo pathway can allow LECs to persist into adulthood. We have also identified a developmental window in which Yki translocates to the nucleus in LECs and have correlated this to an increase in the expression of a known Hippo pathway target gene, *Diap1*. Combining these results, we have revealed a role for the Hippo pathway in regulating the timing of LEC clearance during abdominal development. Our model for this role is presented in Fig. 8. Furthermore, these findings clarify the previously reported tumorigenic effect of downregulating Hippo signalling in the abdomen (Mok and Choi, 2022); we show that these growths are persistent LECs rather than tumours caused by excessive cellular growth and/or proliferation.

While we have identified a role for Hippo signalling in LEC clearance, the cues involved in controlling the pathway during this process are yet to be identified. From our results, it appears that a developmental event is occurring around 14 h APF to induce Yki to enter the nucleus and drive the expression of pro-survival genes. Furthermore, our finding that Yki is predominantly cytoplasmic at 26 h APF suggests that another cue is acting later in development to draw Yki back out of the nucleus, promoting LEC apoptosis. Changes in mechanical tension during abdominal development may be one factor influencing Hippo pathway activity throughout this process. For example, extracellular matrix compaction occurring shortly after head eversion (Davis et al., 2022) may be altering substrate stiffness, triggering Yki to enter the nucleus and induce the expression of pro-survival genes (Dupont et al., 2011). Progressive extracellular matrix degradation throughout abdominal development (Davis et al., 2022) could then act to draw Yki back out of the nucleus, leading to an induction of apoptosis (Zhao et al., 2012; Fraire-Zamora et al., 2021). Given previous findings for mammalian YAP (Zhao et al., 2007), changes in cell density and morphology due to histoblast nest expansion (Bischoff, 2012) may also be promoting the cytoplasmic localization of Yki during the latter stages of abdominal development. The finding that Yki is predominately cytoplasmic in pre-apoptotic LECs expected to be experiencing heightened junctional tension (Michel and Dahmann, 2020) conflicts with previous reports that elevated junctional tension actually decreases Hippo pathway activity (Rauskolb et al., 2014; Tokamov et al., 2023), and that cells experiencing high tension are less likely to be extruded in the notum (Curran et al., 2017). It should be noted, however, that these studies were performed using actively dividing columnar cells originating from the wing disc. Given that LECs are non-dividing squamous cells, it is difficult to say whether the normal conventions of the Hippo pathway are applicable in this cell type. Further research is needed to accurately understand how mechanical tension affects Hippo pathway function in LECs.

A role for ecdysone signalling also seems plausible given its well-established role in developmental cell death (Nicolson et al., 2015). The clearance of LECs during abdominal development has been shown to be dependent on ecdysone signalling (Ninov et al., 2007), with LEC persistence being partially rescued through simple ecdysone feeding assays (Sekyrova et al., 2010; Sztal et al., 2012). The mechanisms by which ecdysone signalling influences LEC survival are poorly understood. Work in *Helicoverpa armigera* has indicated that the Hippo pathway responds to ecdysone signalling during development to direct programmed cell death (Dong et al., 2015; Wang et al., 2016). Whether this relationship between ecdysone and Hippo signalling is maintained in *Drosophila* has, however, not yet been assessed.

From a morphological perspective, the manner in which LECs persist in a Hippo pathway-downregulated background is unique. Multiple studies have demonstrated LEC survival into adulthood (Madhavan and Madhavan, 1984; Sekyrova et al., 2010; Yuswan et al., 2024), but in each of these cases persisting LECs remain in line with the adult epithelium, producing phenotypes primarily associated with defective cuticle formation (Kester and Nambu, 2011). In comparison, when the Hippo pathway is downregulated, persisting LECs do not appear to remain in line with the adult epithelium, instead forming mounds of circular masses that protrude apically above the adult cuticle. The shape of these LECs is reminiscent of structures formed by *wts* and *hpo* mutant clones in other tissues (Justice et al., 1995; Wu et al., 2003). These cell shape changes may be due to apical hypertrophy occurring in Hippo pathway-downregulated LECs, as has been demonstrated in other cell types due to an expansion of the apical domain associated with the aberrant accumulation of a variety of membrane proteins (Genevet et al., 2009). Given that LECs are known to undergo apical constriction to facilitate their basal extrusion during abdominal morphogenesis (Bischoff, 2012), it may be worthwhile to explore the effect apical hypertrophy in assisting Hippo pathway-downregulated LECs to persist into adulthood.

From our results, it appears that *Diap1* and *ban* are two Hippo pathway target genes able to induce LEC persistence. An increase in *Diap1* expression is potentially influencing LEC clearance by blocking caspase activation during development (Kester and Nambu, 2011), but the mechanism by which *ban* is affecting LEC survival is less clear. Increased *ban* expression may simply be further enhancing the ability of Diap1 to block caspase activation through the previously reported regulatory effect of this microRNA on *hid* (Brennecke et al., 2003). However, LECs experiencing a blockage in caspase activation through overexpression of *Diap1* or the baculovirus protein p35 still undergo basal extrusion, surviving beneath the adult epithelium rather than within it (Ninov et al., 2007). Upon *wts* knockdown, the basal extrusion of LECs appears to be incomplete, indicating that additional processes other than caspase activation may be disrupted. Recent research has identified reduced *ban* expression as a key factor underlying the high propensity of *yki* null clones to undergo extrusion in the wing disc (Ai et al., 2020). Therefore, *ban* may be acting synergistically with Diap1 to drive LEC persistence, preventing LEC extrusion while Diap1 blocks caspase activation. Additional experiments would, however, be required to test this notion.

Our finding that Hippo signalling regulates LEC clearance in a Sd-dependent manner is a noteworthy example of the role of this pathway in a normal developmental context. While the ability of Hippo signalling to regulate apoptosis is well established (Huang et al., 2005; Udan et al., 2003; Harvey et al., 2003), considerably more attention has been directed towards the growth-promoting role of the pathway. Recently, there has been increasing debate about the relevance of the growth-promoting role of the Hippo pathway during normal development (Kowalczyk et al., 2022), since Sd-mediated transcription appears to be expendable for growth in a variety of tissues (Guo et al., 2013; Koontz et al., 2013). In contrast, Sd-mediated transcription plays a role unrelated to growth during abdominal development, instead being important for coordinating the correct timing of LEC clearance (Fig. 8). The expression of pro-survival genes during early abdominal development appears to prevent the premature induction of apoptosis in LECs, ensuring the integrity of the epithelium is maintained in the period before histoblast nest expansion. Downregulating the initial expression of these pro-survival factors, either by upregulating Hippo signalling or downregulating *sd*, results in the early removal of LECs from the epithelium, disrupting histoblast migration and disfiguring the resultant adult abdomen. In comparison, maintaining or heightening the expression of pro-survival genes by downregulating the Hippo pathway promotes the continued survival of LECs, preventing their appropriate removal from the epithelium. The clearance of larval trachea and salivary glands has been shown to be responsive to changes in Hippo pathway activity (Fraire-Zamora et al., 2021; Dutta and Baehrecke, 2008). Recent work has likewise shown that Hippo signalling regulates apoptosis in the pupal thorax (Cachoux et al., 2023), and there has been speculation that the Hippo pathway may regulate developmental cell death in the amnioserosa (Gorfinkiel et al., 2025). In many of these instances, the transcription of pro-survival genes is associated with this regulation of apoptosis. As such, it may be interesting to consider whether this function of Hippo signalling during LEC clearance is indicative of a broader role for the pathway in terms of regulating epithelial cell replacement during morphogenesis.

## MATERIALS AND METHODS

### Fly stocks and husbandry

The following fly stocks were used in this study: *w^1118^* [BL (Bloomington *Drosophila* Stock Center) 3605], *pannier-GAL4* (BL3039), *Eip71CD-GAL4* (BL6871), *UAS-Apoliner* (BL32122), *UAS-Diap1* (BL6657), *UAS-EGFP-mir-ban* (BL60672), *UAS-Myc* (BL9674), *UAS-CycE* (BL4781), *UAS-yki^S168A^* (BL28818), *yki-Venus* (BL604911), *tubGAL80^ts^; pannier-GAL4* (BL67060), *His2Av-mRFP* (BL23651), *UAS-GC3Ai* (BL84346), *Diap1-4.3-GFP* (Zhang et al., 2008), *ban-3-GFPnls* (Matakatsu and Blair, 2012), *UAS-His2A-RFP* (Emery et al., 2005), *UAS-sd^RNAi^* (Zhang et al., 2008), *UAS-hpo* (Udan et al., 2003), *UAS*-*dsRed*-*bantam*-*sponge* (Becam et al., 2011). *UAS-wts^RNAi^* (stock #111002) and *UAS-lacZ^RNAi^* (stock #51446) were obtained from the Vienna *Drosophila* Resource Center.

Flies were reared on standard medium at 25°C unless stated otherwise in the text. For temperature-shift experiments, vials were placed at 18°C until pupae started to develop, at which point pupae were shifted to 29°C for the duration of pupal development.

### Live imaging

Imaging was performed as described previously (Ninov and Martín-Blanco, 2007). Staged pupae were obtained by taking white prepupae (0 h APF) and ageing them at specified temperatures. For brown prepupae, which had already passed the white prepupal stage, pupae were taken and placed in a dish where they were checked hourly for signs of head eversion (12 h APF). Appropriately staged pupae were then placed dorsal side up on a piece of double-sided tape and their pupal cases removed. Between imaging steps, pupae were kept in an enclosed dish containing a piece of moistened paper towel and placed into an incubator to maintain the desired developmental temperature. For adult imaging, flies were anaesthetized with CO_2_ and their wings and legs were removed to allow for visualization of the dorsal and ventral abdomen. Brightfield and fluorescence images were taken in Leica Application suite (v4.12) software using a Leica M165 FC Fluorescent Stereo Microscope with a Leica DFC450 C digital camera. Abdominal *Diap1*-4.3-GFP intensities were measured as mean grey values using ImageJ (v1.53 K). Images depict *z*-stacks merged using Zerene Stacker (v1.04) software.

For confocal imaging, pupae mounted on double-sided tape were attached dorsal side down to a 35 mm glass-bottom dish (Cellvis, D35-20-1.5H) such that their abdomen made light contact with the coverslip. Pieces of damp paper towel were placed around the edges of the dish to maintain humidity. Imaging was performed using an inverted confocal laser-scanning microscope (Zeiss LSM980) with either 10× or 20× objectives. *Z*-stacks of abdominal segment A3 were collected for each abdomen at each indicated time point.

### Image and data analysis

LEC nuclei in the A3 segment of pupae were manually counted using the multi-point tool in ImageJ at each indicated time point. Condensed nuclei and fluorescent puncta associated with cellular debris were excluded from counts. Higher magnification views of A3 segments were subjected to a rolling ball background subtraction (radius 100 pixels) using the ‘Subtract Background’ function in ImageJ to enhance nuclei visibility. All analysis was performed on the original unedited images. Measurements of *Diap1*-4.3-GFP intensity were generated in ImageJ by creating a region of interest encompassing the entirety of the pupal abdomen and measuring the mean grey value of each pupa at each time point. Proportions of cells displaying nuclear Apoliner GFP were determined by thresholding images of Apoliner RFP fluorescence to first gain counts of total nuclei numbers in a segment, followed by thresholding of Apoliner GFP images to detect cells which displayed levels of nuclear fluorescence above cytoplasmic signal. Measurements of the nucleocytoplasmic distribution of Yki-Venus localization were performed by segmenting the nuclei using His2A-RFP fluorescence and subsequently measuring fluorescence intensity within these nuclei compared to the inverse across the remainder of the image.

### Statistical analysis

All statistics and graphs were generated using R studio with R version 4.2.1. *P*<0.05 was taken to be a statistically significant difference. Comparisons were made between ratios of nuclear-to-cytoplasmic Yki-Venus localization using an unpaired two-tailed Student's *t*-test. Linear regression models were fitted to measurements of *Diap1*-4.3-GFP fluorescence, with the slope of these linear regressions being compared with Welch's *t*-tests. LEC nuclei count distributions were compared using a Kolmogorov–Smirnov test. The proportion of nuclei displaying Apoliner GFP fluorescence was compared using an unpaired two-tailed Student's *t*-test.

## Supplementary Material



10.1242/joces.264798_sup1 Supplementary information
