# Supplementary material for: The Hippo signalling pathway regulates the timing of epithelial cell replacement during Drosophila abdominal morphogenesis
Source: J Cell Sci. 2026 Sep 15;139(17):jcs264798. doi: 10.1242/jcs.264798 (PMC13615693; doi:10.1242/jcs.264798)
Supplement: Supplementary file 1 [file joces-139-264798-s1.pdf]

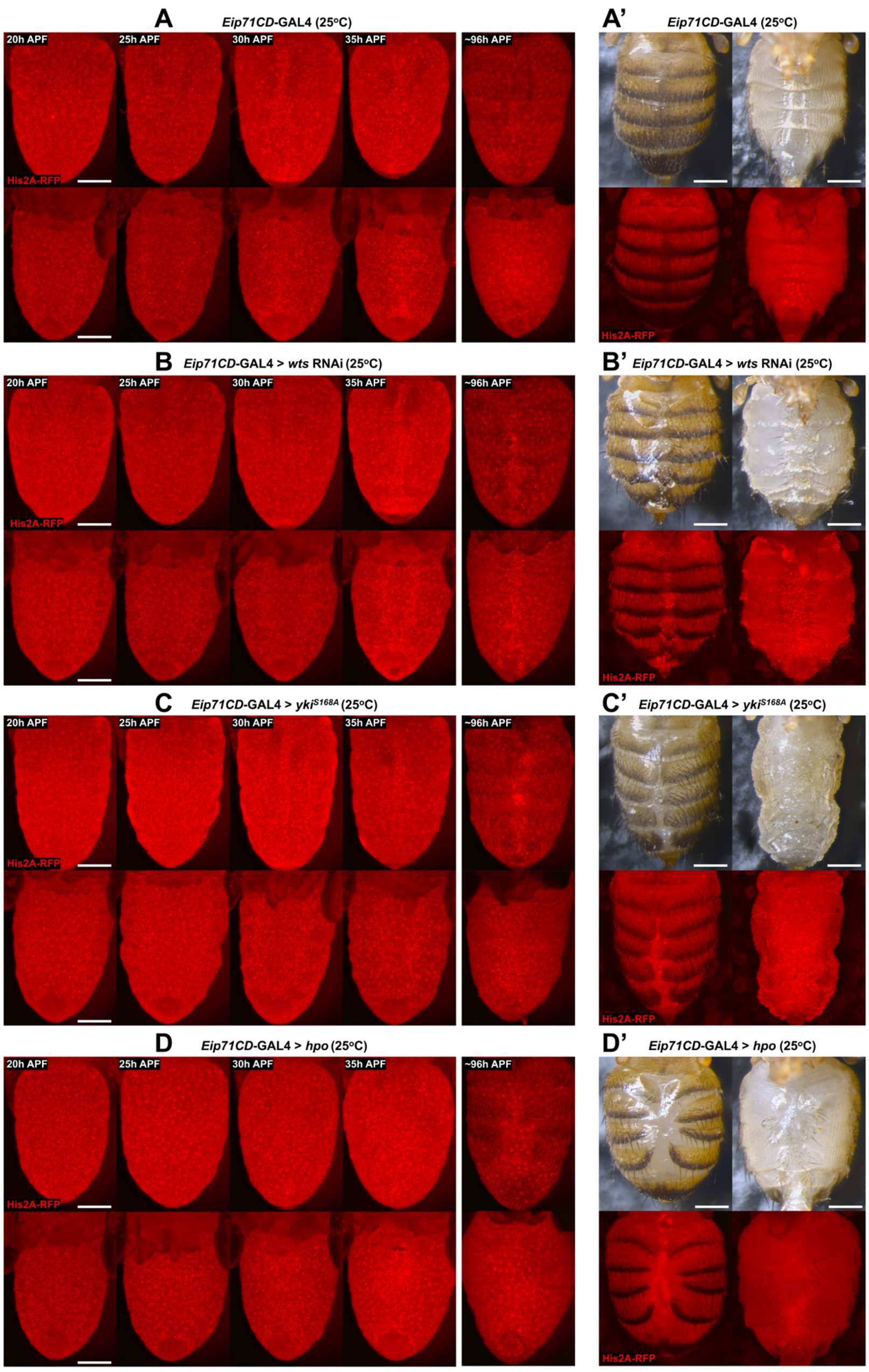


**Fig. S1. Altering the expression of Hippo pathway components under *Eip71CD*-GAL4 induces a phenotype associated with LEC persistence.** (**A-D**) Staged pupae and (**A'-D'**) adult flies expressing a UAS-responsive His2A-RFP under *EIp71CD*-GAL4 in the following backgrounds: (**A**, **A'**) control ($w^{1118}$), (**B**, **B'**) *wts* RNAi, (**C**, **C'**) $yki^{s168A}$ overexpression or (**D**, **D'**) *hpo* overexpression. (**A-D**) The strong expression of *Eip71CD*-GAL4 in the fat body obfuscates viewing the progression of LEC clearance during the pupal stage using a UAS-responsive fluorescent protein. (**A'**) No LECs are present in the abdomen of control flies, with slight His2A-RFP fluorescence visible along the sternites believed to be associated with expression in the fat body. (**B'**) LEC nuclei can be seen in the clumps of protruding tissue present in the abdomen of pupae experiencing *wts* RNAi under *Eip71CD*-GAL4. (**C'**) The ventral abdomen of $yki^{S168A}$ overexpression adults contains a considerable number of persistent LECs. (**D'**) No LECs are visible in the cuticle clefts induced by *hpo* overexpression. Scale bars: 250 µm.

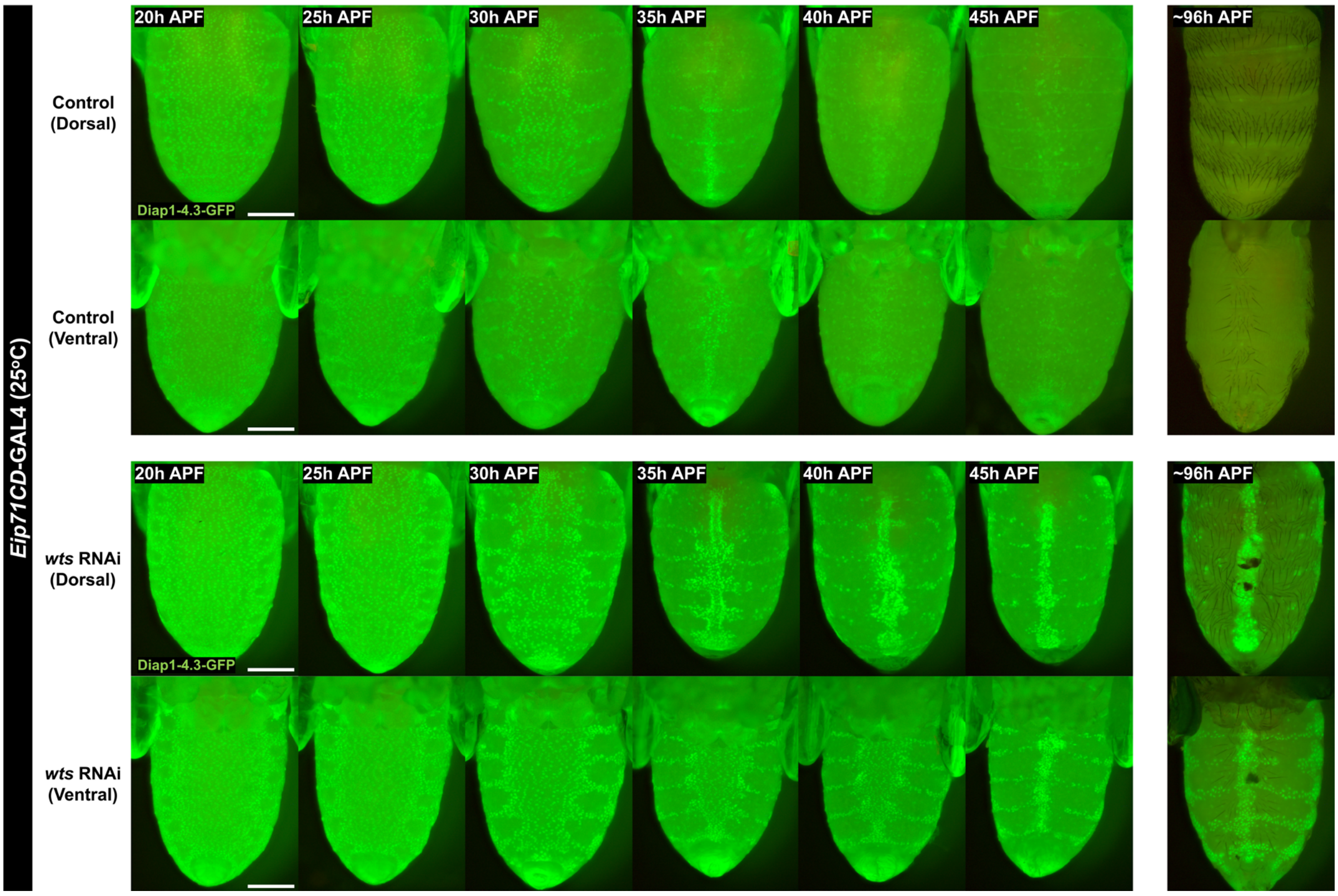


**Fig. S2. Knocking down *wts* under *Eip71CD*-GAL4 disrupts the progressive clearance of LECs, causing LECs to persist into adulthood** Dorsal (top) and ventral (bottom) views of pupae expressing the transcriptional reporter *Diap1*-4.3-GFP in a control ($w^{1118}$) background or in the presence of *wts* RNAi driven by *Eip71CD*-GAL4. When the Hippo pathway is downregulated through knockdown of the upstream kinase *wts*, LECs survive into adulthood in both the dorsal and ventral sides of the abdomen. Scale bars: 250 µm.

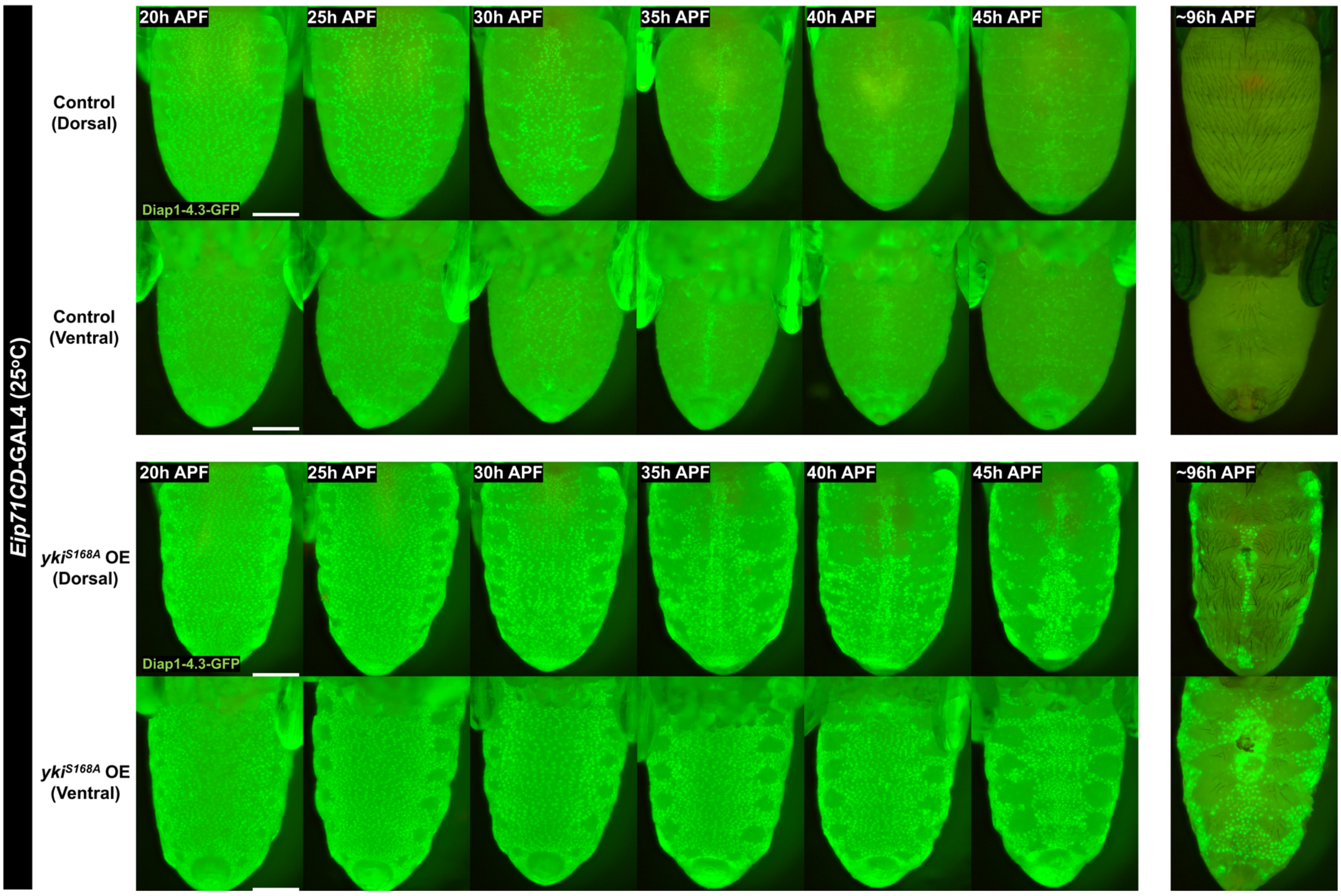


**Fig. S3. Overexpression of a constitutively active form of Yki induces extreme LEC persistence in the ventral abdomen.** Dorsal (top) and ventral (bottom) abdomen from control ($w^{1118}$) pupae and pupae overexpressing a constitutively active version of Yki ($Yki^{S168A}$) under *Eip71CD*-GAL4 in the presence of *Diap1*-4.3-GFP. Heavy LEC persistence can be seen in the abdomen of these animals, particularly on the ventral side. Scale bars: 250 µm.

Journal of Cell Science • Supplementary information

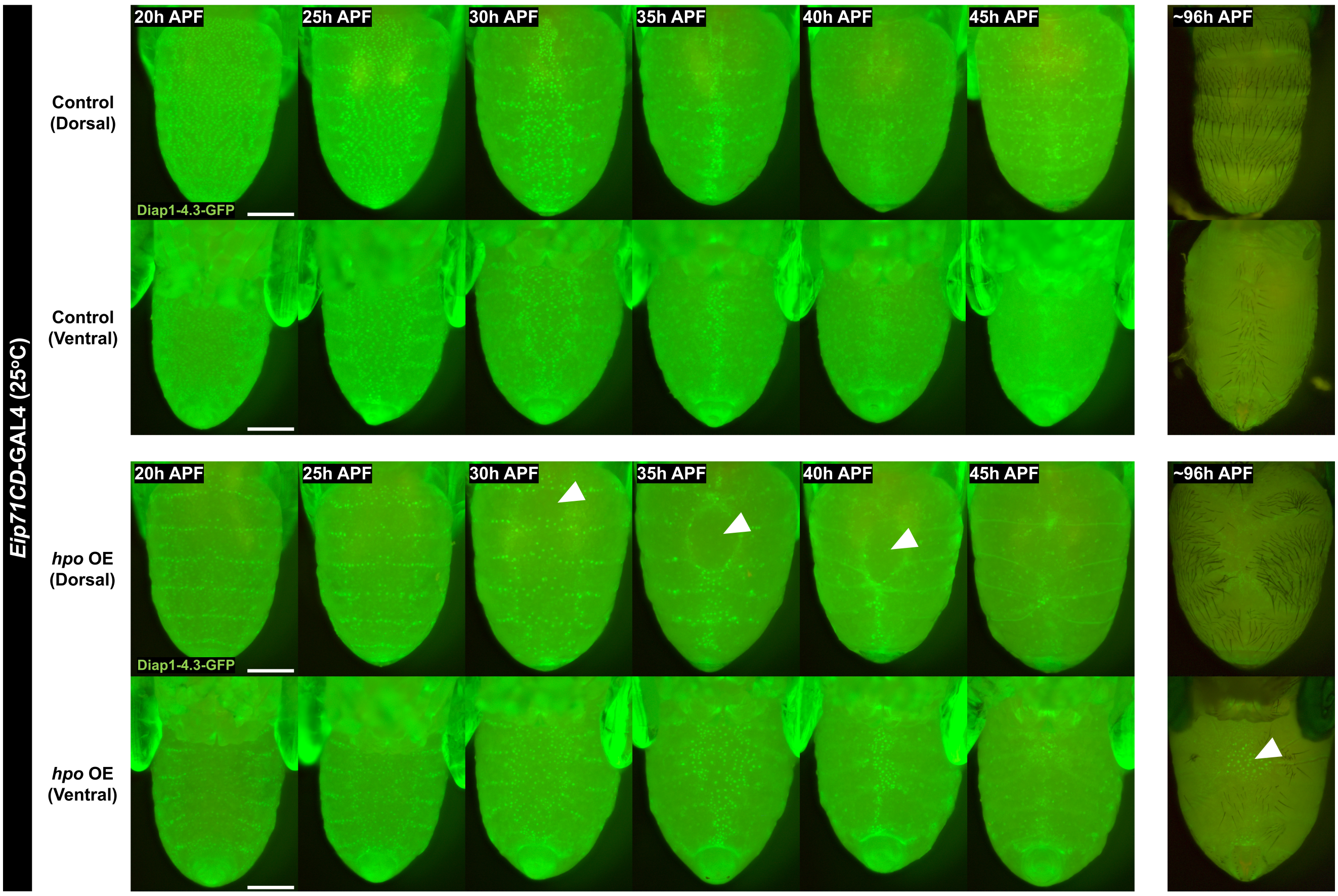


**Fig. S4. Upregulating the Hippo pathway induces early LEC clearance.** Dorsal (top) and ventral (bottom) abdomen of control (*w*[1118]) flies and flies overexpressing *hpo* under *Eip71CD*-GAL4 in the presence of *Diap1*-4.3-GFP. Overexpressing *hpo* strongly downregulates *Diap1*-4.3-GFP expression in LECs. This loss of *Diap1*-4.-3-GFP expression appears to result in premature LEC apoptosis, as gaps frequently form in the abdomen of these pupae between 30-35 h APF (white arrowheads). Pharate adults overexpressing hpo commonly have disfigured abdomen, with the occasional appearance of LEC nuclei believed to be associated with persistent tendon cells (~96 h APF white arrowhead). Scale bar: 250 µm.

Journal of Cell Science • Supplementary information

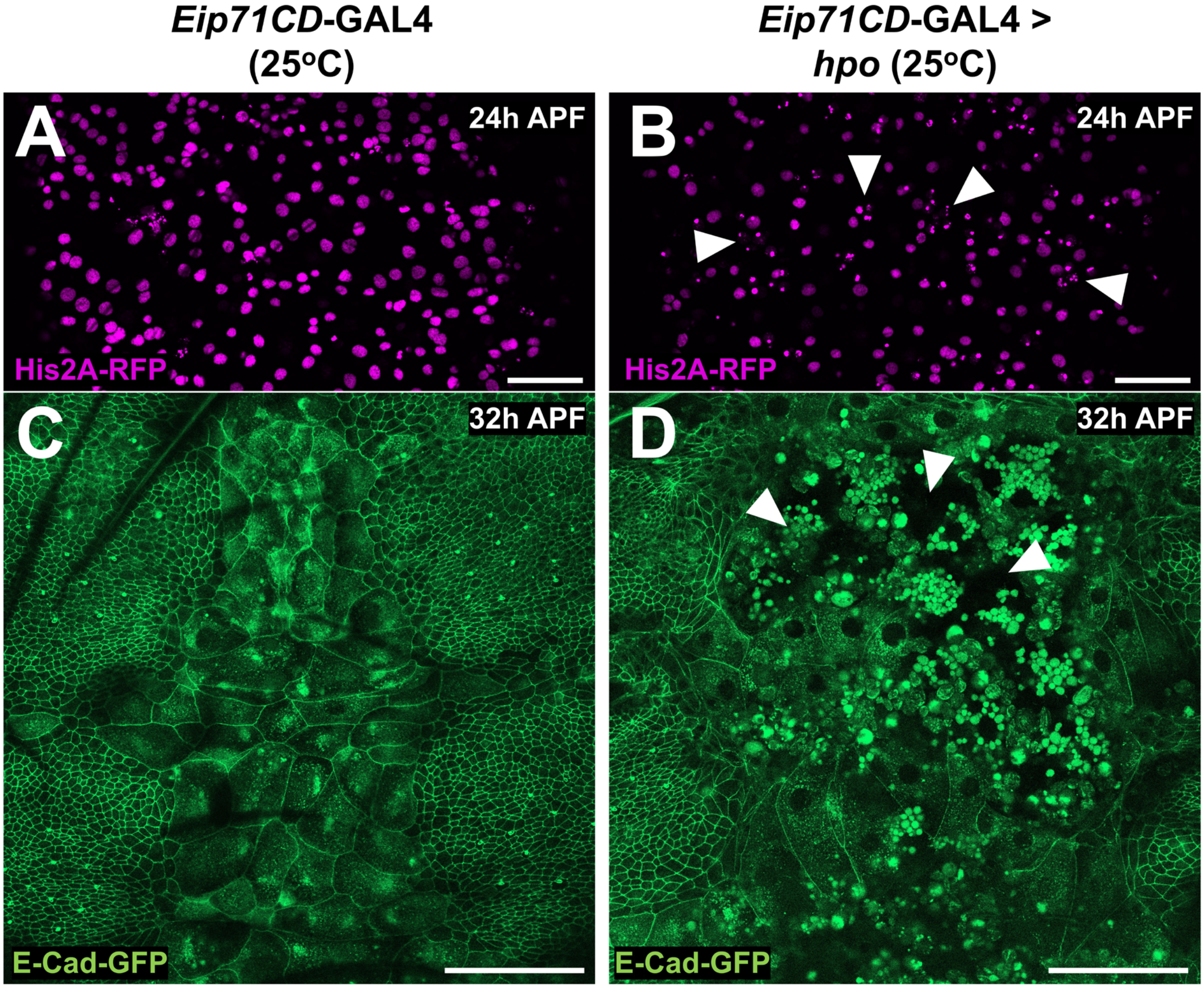


**Fig. S5. Overexpressing *hpo* in pupal LECs induces their premature apoptosis, resulting in a disruption of epithelial integrity.** Dorsal abdomen of staged pupae expressing (**A**, **B**) a UAS-responsive His2A-RFP under *Eip71CD*-GAL4 or (**C**, **D**) an endogenously tagged E-Cadherin(E-Cad)-GFP in a (**A**, **C**) control or (**B**, **D**) *hpo* overexpression background. (**A**) At 24 h APF the majority of LEC nuclei in a control background are intact. (**B**) LECs overexpressing *hpo* at the same time point frequently display signs of nuclear condensation and loss of nuclear architecture believed to be associated with an induction of apoptosis (white arrowheads). (**C**) At 32 h APF, histoblasts and LECs typically form a contiguous epithelial layer spanning the entirety of the abdomen. (**D**) Overexpressing *hpo* in LECs disrupts this epithelial layer, with large regions containing neither histoblasts nor LECs being apparent at 32 h APF (white arrowheads). Scale bars: 100 µm.

Journal of Cell Science • Supplementary information

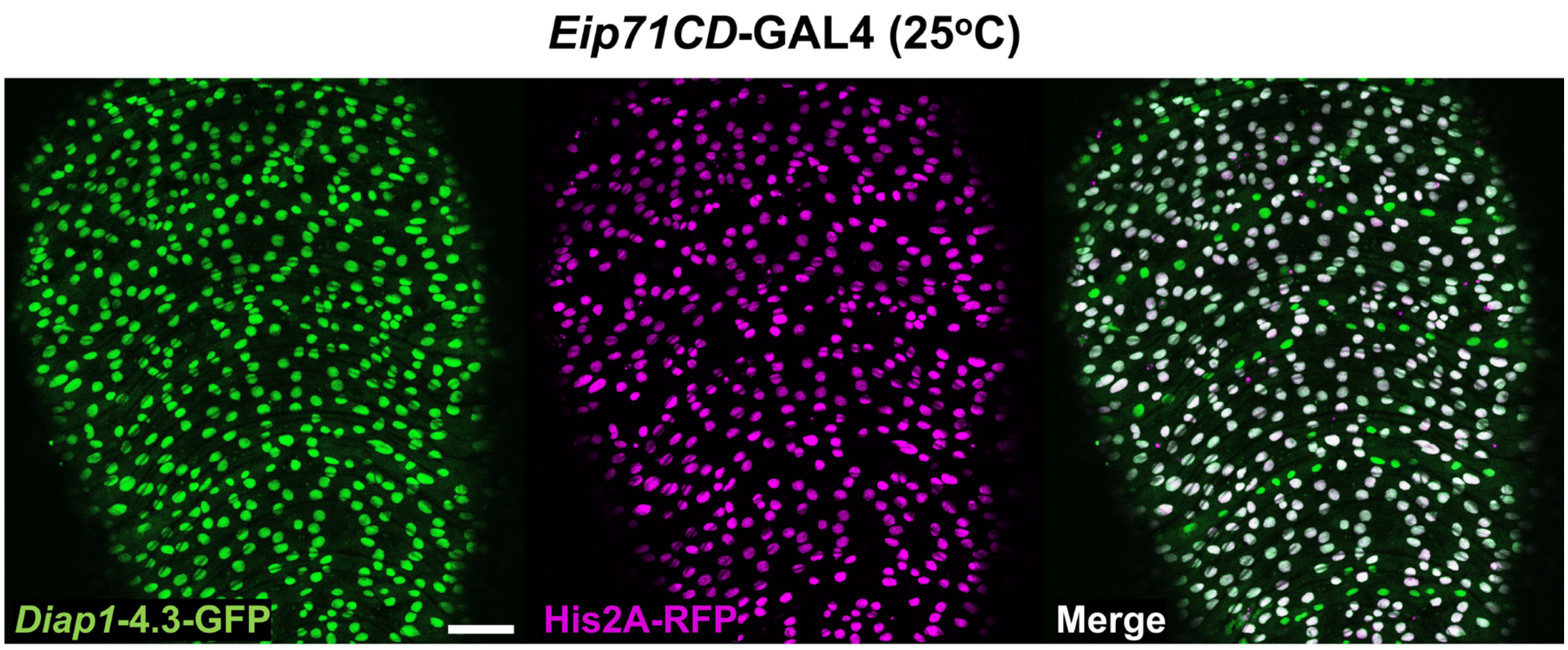


**Fig. S6. The GAL4 driver *Eip71CD*-GAL4 does not drive expression in abdominal tendon cells.** Early pupal abdomen expressing both *Diap1*-4.3-GFP and a UAS-responsive His2A-RFP under *Eip71CD*-GAL4. In the developing abdomen, *Diap1*-4.3-GFP is ubiquitously expressed in both segmental LECs and tendon cells. When driving His2A-RFP expression under *Eip71CD*-GAL4, gaps in the expression of the GAL4 driver can be seen in the abdominal epithelium. These gaps appear to correspond to the segmental boundaries in which tendon cells are found. Scale bar: 100 µm.

Journal of Cell Science • Supplementary information

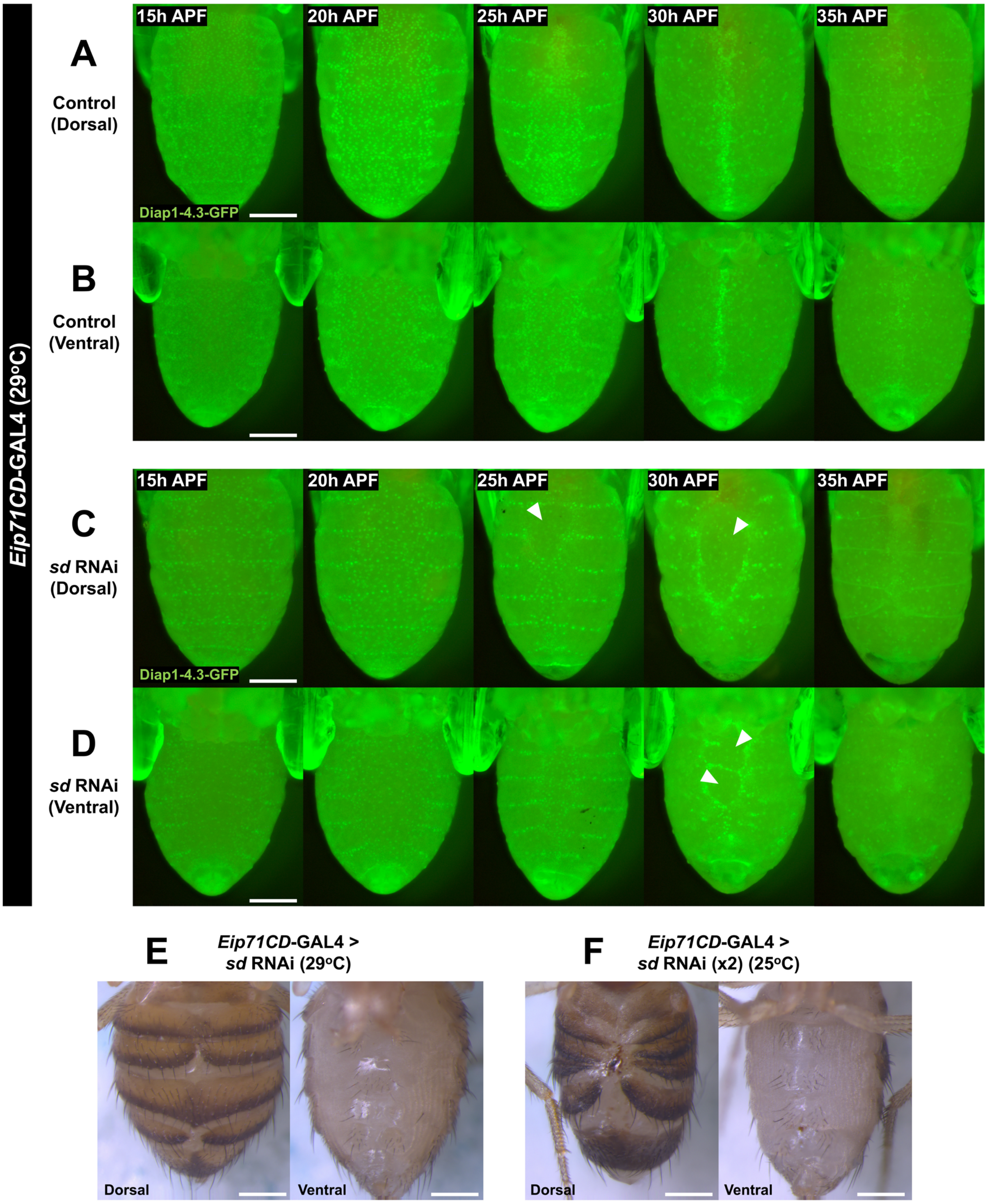


**Fig. S7. Downregulating *sd* in LECs disrupts the formation of the adult abdomen.** (**A, C**) Dorsal and (**B**, **D**) ventral views of pupal abdomen from control flies or flies expressing *sd* RNAi under *Eip71CD*-GAL4 in the presence (**A**) of the transcriptional reporter *Diap1*-4.3-GFP. The resultant adult abdominal phenotypes of flies expressing (**E**) one copy of *sd* RNAi at 29ºC or (**F**) two copies at 25ºC. (**A**) The progression of abdominal morphogenesis in a control animal between 15-35 h APF. (**B**) Knocking down *sd* under *Eip71CD*-GAL4 strongly downregulates *Diap1* expression in segmental LECs at 29ºC. As development progresses, *sd* knockdown also induces holes to form in the developing epithelium (white arrowheads). (**E**) Adult *sd* knockdown flies raised at 29ºC display clefts in the dorsal cuticle as well as slight disruptions in the location of sternal bristles. (**F**) Flies homozygous for *sd* RNAi display prominent defects in their dorsal abdomen at 25ºC. Scale bars: 250 µm.

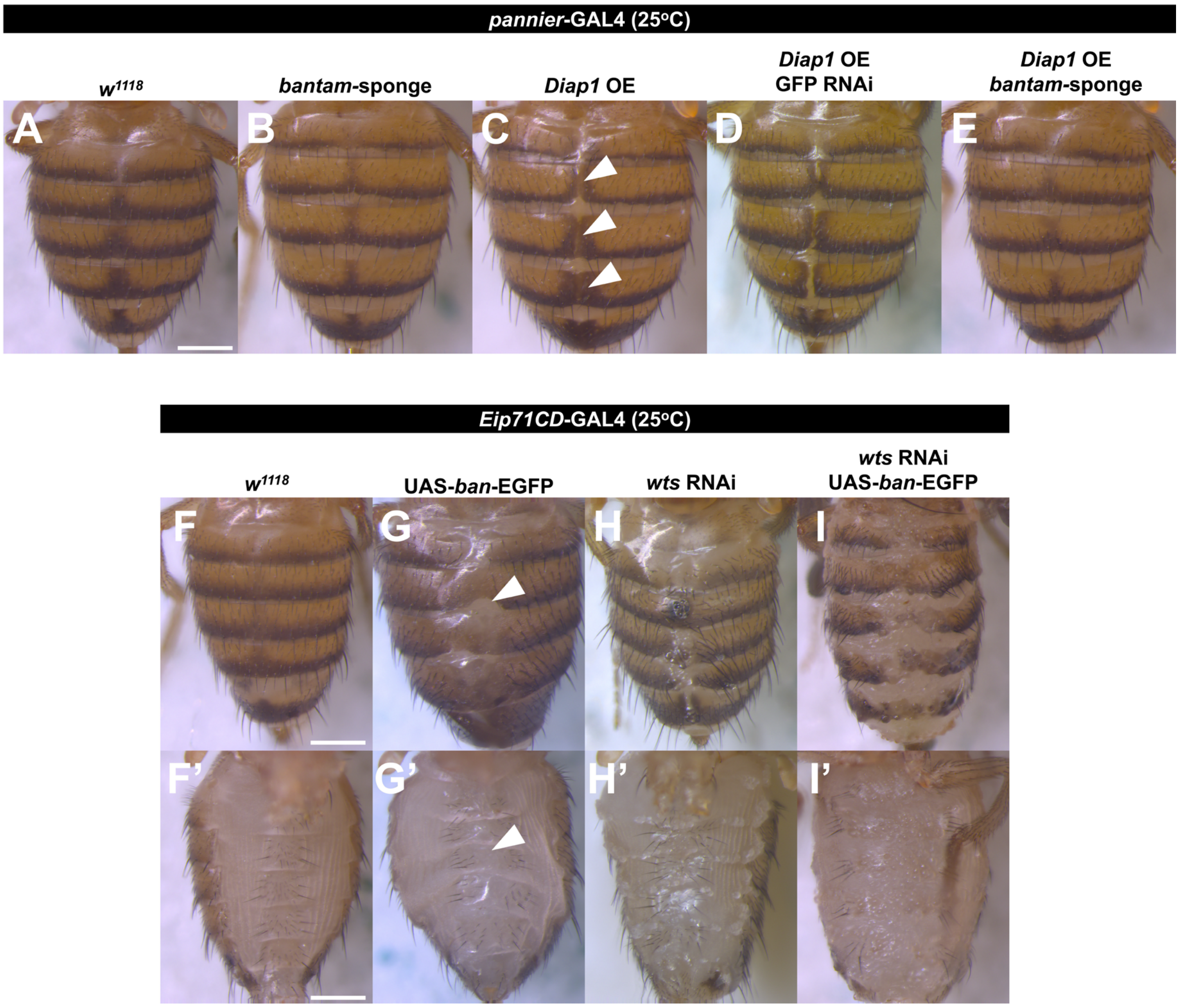


**Fig. S8. Altering the expression of *ban* influences phenotypes associated with disrupted LEC clearance.** (**A**-**E**) The adult abdomen of flies expressing combinations of *Diap1* and *bantam*-sponge under *pannier*-GAL4. (**F**-**I**) Dorsal and ventral abdomen of flies expressing *wts* RNAi and *ban* either alone or in combination under *Eip71CD*-GAL4. (**B**) *bantam*-sponge has no phenotypic effect on its own under *pannier*-GAL4. (**C**) Overexpressing *Diap1* induces a cuticular split along the midline of the dorsal abdomen (white arrowheads). (**D**) The addition of a second UAS element has no effect on this phenotype. (**E**) Downregulating *ban* expression completely suppresses the phenotypic effects of *Diap1* overexpression. (**G, G'**) Under *Eip71CD*-GAL4, *ban* overexpression induces a mottled discolouration of the dorsal cuticle and a partial displacement of the sternites in the ventral abdomen (white arrowheads). (**I, I'**) LEC persistence appears to be enhanced when *ban* overexpression is combined with *wts* RNAi, with persistent LECs appearing to remain contiguous with the surrounding epithelium. All crosses were performed at 25°C. Scale bars: 250 µm.

Journal of Cell Science • Supplementary information

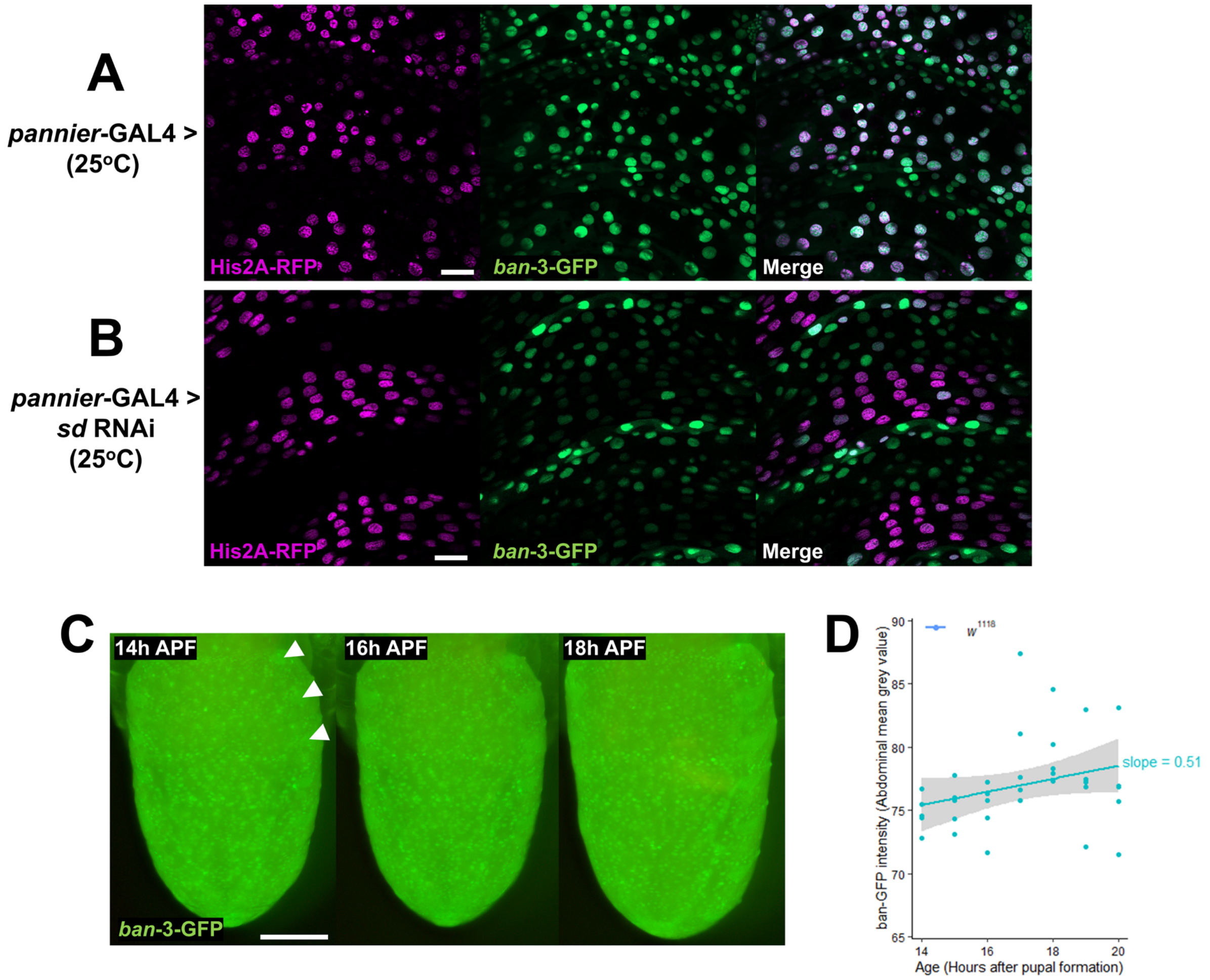


**Fig. S9. The Hippo-target gene *bantam* is endogenously expressed in LECs.** The dorsal abdomen of flies expressing the transcriptional reporter *ban*-3-GFP (**C**) and a UAS-responsive His2A-RFP in the presence (**B**) or absence (**A**) of *sd* RNAi expression under *pannier*-GAL4. (**A**) The transcriptional reporter *ban*-3-GFP indicates that *ban* is endogenously expressed in both LECs and histoblasts (white arrowheads indicate examples of histoblast *ban* expression at 14 h APF). (**B**) Knocking down *sd* reduces ban expression in *pannier* expressing LECs. (**C**) The expression of *ban* in LECs does not appear to change considerably during the early stages of abdominal development. (**D**) Plot depicting *ban*-3-GFP intensities in the abdomen between 14-20 h APF, with there being no significant relationship between *ban*-3-GFP intensity and age across these time points ($P$ = 0.08, Linear regression model). $n$ = 5 pupae at each time point. Time points indicate hours after puparium formation. Scale bars: 100 µm in A,B and 250 µm in C.
