# Supplementary material for: The Hippo signalling pathway regulates the timing of epithelial cell replacement during Drosophila abdominal morphogenesis
Source: J Cell Sci. 2026 Sep 15;139(17):jcs264798. doi: 10.1242/jcs.264798 (PMC13615693; doi:10.1242/jcs.264798)
Supplement: Supplementary file 2 [file joces-139-264798_review_history.pdf]

Journal of Cell Science

The Hippo signalling pathway regulates the timing of epithelial cell replacement during *Drosophila* abdominal morphogenesis

Kyle Lyon, Samuel A. Manning and Richard Burke
DOI: 10.1242/jcs.264798

Editor: Richa Rikhy

Review timeline
Original submission: 15 February 2026
Editorial decision: 11 March 2026
First revision received: 8 June 2026
Editorial decision: 5 July 2026
Second revision received: 27 July 2026
Accepted: 31 July 2026

Original submission

First decision letter

MS ID#: jcs.264798

MS TITLE: The Hippo signalling pathway regulates the timing of epithelial cell replacement during *Drosophila* abdominal morphogenesis

AUTHORS: Kyle Lyon; Samuel A. Manning; Richard Burke

ARTICLE TYPE: Research Article

Dear Dr Burke,

We have now reached a decision on the above manuscript.

As you will see, the reviewers raise a number of substantial criticisms that prevent me from accepting the paper at this stage. They suggest, however, that a revised version might prove acceptable, if you can address their concerns. If you think that you can deal satisfactorily with the criticisms on revision, I would be pleased to see a revised manuscript. We would then return it to the reviewers.

Reviewer 1

SUMMARY OF THE ADVANCE MADE IN THIS PAPER AND ITS POTENTIAL SIGNIFICANCE TO THE FIELD

In this study, the authors investigated the signaling pathways involved in the coordinated regulation of apoptosis in larval epidermal cells (LECs) and the proliferation of presumptive adult epithelial cells during the development of the adult fly abdomen. They found that wts-RNAi in LECs disrupted abdominal development. Abdominal outgrowths caused by Hippo pathway downregulation occurred due to a disruption in LEC clearance. sd-RNAi in LEC increased the rate of LEC apoptosis, indicating that Hippo pathway dependent transcription is important for LEC survival. Temporal changes in Diap1 expression depended on the Hippo pathway in LEC clearance and were correlated with nuclear Yki localization. The authors suggested that Yki enters the nucleus in LECs during the early stages of abdominal development, potentially allowing for the Hippo pathway to direct the

© 2026. Published by The Company of Biologists under the terms of the Creative Commons Attribution License (https://creativecommons.org/licenses/by/4.0/).

transcription of Diap1. wts-RNAi in LECs resulted in a reduction in apoptosis, which was also correlated with nuclear Yki localization. Overexpression of Diap1 or ban also resulted in the formation of prominent cuticular clefts along the length of the dorsal abdomen. The authors also concluded that Diap1 and ban are two target genes of the Hippo pathway that potentially influence wts-RNAi-induced LEC survival. Overall, the authors proposed that the downregulation of Hippo signaling in LECs promotes their survival into adulthood, while the upregulation of Hippo signaling induces their premature removal from the epithelium.

The manuscript is well written, and the results are clearly presented. This finding clearly demonstrates a role of Hippo signaling in regulating LEC death during abdominal morphogenesis. I have a few relatively minor comments, explained below.

Minor comments

- All figures where arrowheads are shown: The arrowheads should be enlarged and colored in against a nearly white background.

-Line 135-139: The authors showed that the defect in LEC apoptosis is caused by downregulation of the Hippo pathway. However, they did not investigate whether histoblast overgrowth (or proliferation?) is also caused by the Hippo pathway downregulation in this study. The authors should comment on this.

-Line 226: Correct (Fig. 5E) to (Fig. 5F and H).

-Line 226: (Fig. 5F) to (Fig. 5G).

-Line 227: (Fig. 5G) to (Fig. 5H).

-Line 279-280: The authors should provide a brief explanation of 'bantam-sponge'.

<u>Reviewer 2</u>

SUMMARY OF THE ADVANCE MADE IN THIS PAPER AND ITS POTENTIAL SIGNIFICANCE TO THE FIELD

The manuscript by Lyon et colleagues addresses the role of Hippo signaling in the removal of larval epidermal cells (LECs) from the Drosophila abdominal epidermis during pupal development. The authors show that knockdown of Hippo signaling impairs LEC removal, whereas overexpression of hippo results in premature LEC removal. Further, the authors provide evidence that Yorkie, a negative target of Hippo signaling, accumulates in the nucleus during early pupal development and, through the co-transcriptional activator Sd, induces the expression of genes promoting cell survival. The manuscript reports a novel and interesting role of Hippo signaling during cell replacement in Drosophila. In general, the manuscript is well written and conclusions are well supported by data. However, there are a number of issues the authors should address.

SUGGESTIONS TO AUTHORS

Major comments
Fig. 2A,C The authors state that LECs are completely cleared by 35h APF, however, there seems to be a substantial number of LEC nuclei still present at that time point (in particular at the dorsal midline). The images do not seem to represent the quantification for this time point. The authors should clarify.

Fig. 2A,B The authors show images of the multiple segments of the dorsal abdomen, but analyze only segment A3. To increase clarity of the data, the authors should show (in addition) higher magnification views focusing on segment A3.

Fig. 3B,S1 The authors claim that knockdown of wts using the Eip71CD-Gal4 driver results in LEC persistence. This conclusion has two limitations: First, Fig. 3B does not identify LECs, but shows brightfield images of the abdomen. Second, Fig. S1 does identify LECs, but only those expressing Diap1-GFP. We therefore cannot distinguish whether LECs no longer express the Diap1-GFP reporter

© 2026. Published by The Company of Biologists under the terms of the Creative Commons Attribution License (https://creativecommons.org/licenses/by/4.0/).

or whether the LECs have been cleared. The number of cells that are not cleared might therefore be under-estimated. The authors should show fluorescence images of Eip71CD-Gal4 driving a nuclear marker and wts knockdown. Moreover, Fig. S1 is missing a negative control. The authors should therefore also show fluorescence images of Eip71CD-Gal4 driving a nuclear marker (without wts knockdown). Staging should preferably be h AEL (it is unclear what 'visible developmental features' are (legend to Fig. S1).

Fig. S2 is missing a negative control. The authors should show fluorescence images of Eip71CD-Gal4 driving a nuclear marker (without Yki-S168A).

Fig. S3, line 175 The authors claim that pupae exhibited gaps in the developing epithelium. These gaps are difficult to see. The authors should show more appropriate images.

Fig. 4B, line 191 The authors claim signs of condensed nuclei and apoptotic cells at 15 h APF in sd knockdowns. The authors should explain how they identify condensed nuclei and apoptotic cells and show higher magnification views to corroborate their findings. Moreover, controls apparently also show condensed nuclei at this time point. The authors should quantify the number of condensed nuclei in both genotypes.

Fig. 5C Knockdown of sd at 25C results in decreased DIAP1 expression, but does not influence LEC removal. Does this mean that DIAP1 expression is not important for LEC removal, which appears to be inconsistent with Kester and Nambu, 2011? The authors should clarify.

Fig. 7A' The authors claim that, when DIAP1 is overexpressed, surviving LECs persist underneath the adult epithelium. This is difficult to see from the shown image. The authors should show better images.

Line 257 The authors claim that LECs persisting following wts knockdown do not extrude basally. The authors should indicate to which figure they refer to.

Fig. 7F The authors link clumps of tissue at the posterior most end of flies induced by DIAP1 and ban co-expression with consequence of wts knockdown (Fig. 3B). This resemblance is unclear and the authors should clarify.

Fig. S7A The authors claim that co-expression of bantam-sponge suppresses the abdominal phenotype induced by DIAP1 overexpression. However, expression of two UAS-genes from a single pannier-Gal4 driver may sequester Gal4 and hence decrease DIAP1 expression. The authors should co-express UAS-lacZ or similar with DIAP1 and test whether the DIAP1-induced abdominal phenotype is present (or suppressed).

Minor comments
Fig. 1. The authors should use A,B,C to label individual images and describe the images in the legend. As it stands, the two images on the right are not mentioned in the legend.

Fig. S3 This figure appears to be insufficiently described in the results part, e.g. the data in panels C and D are not mentioned.

Fig. 5F-H, line 226 The panels in the text relating to Yki subcellular distribution should read F,G,H, not E,F,G

Reviewer 3

SUMMARY OF THE ADVANCE MADE IN THIS PAPER AND ITS POTENTIAL SIGNIFICANCE TO THE FIELD

The manuscript by Lyon et al. investigates the role of Hippo signaling in regulating the survival of abdominal larval epithelial cells (LECs) during pupal metamorphosis. The central finding is that elevated Hippo pathway activity, resulting in reduced nuclear Yorkie, promotes LEC elimination. The study includes systematic manipulation of multiple Hippo pathway components, however, the primary observations are largely consistent with the well-established role of Hippo signaling in controlling cell survival. Previous studies have already demonstrated that overexpression of Diap1

© 2026. Published by The Company of Biologists under the terms of the Creative Commons Attribution License (https://creativecommons.org/licenses/by/4.0/).

enhances LEC survival, making the general conclusion that Hippo activation promotes cell elimination somewhat expected.

SUGGESTIONS TO AUTHORS

Major comments

In my view, the most promising and potentially novel aspect of the study lies in its analysis of the temporal dynamics of Hippo pathway activity during LEC removal, particularly the late-phase onset around 26 hrs APF and the role of bantum. However, the conclusions especially regarding the temporal regulation of LEC elimination require stronger experimental support.

1. The data suggest that LEC numbers in control and wts KD are similar until 25 hrs APF, However, this is difficult to reconcile with the images shown in Figure 2A and 2B, where there appears to be a substantial difference in His2A-RFP signal intensity.

- First, the authors should clarify whether these intensity differences are inherent to the genotypes or due to differences in imaging parameters. Second, within the same sample/segment, there appear to be variations in RFP intensity, which could introduce inaccuracies in automated or threshold-based cell counting.

- Importantly, the manuscript lacks methodological details describing how LEC numbers were quantified. Given that these data form the basis for temporal conclusions, the quantification method must be described in sufficient detail.

- To test cell survival/cell death authors should consider taking direct approach. The Apoliner assay driven by pnr-Gal4 at one time-point is not sufficient on its own to support the conclusions. Cell death should be tested at multiple time points in relevant genotypes, possibly adding Dcp1 staining to avoid potential issues with Apoliner expression levels (RFP in 6A appears differently localized compared with 6B, making it difficult to judge the Apoliner levels).

- A more detailed analysis distinguishing isolated versus clustered cell death, similar to the approach used in Yuswan et al., 2024, would be helpful in determining whether the temporal effects of Hippo signaling preferentially influence a specific mode of LEC elimination.

2. Yki-nuclear localization is shown to change between 13 and 14 hrs APF and then again at 26 hrs. In this regard, I have two points:

- Since, not all LECs die at the same time, I was wondering if the nuclear localization or exclusion of Yki correlates with the cell death. Testing this directly would help clarify the relationship between Yki activity and LEC elimination.

- Yki is shown to be excluded from the nucleus until 14h APF. While authors have discussed plausible mechanisms this temporal regulation, they should also address why cell death levels remain low during the 0-13 hrs APF period, despite apparently high Hippo activity.

Minor comments

- Abstract and elsewhere: Statement: "Downregulating Hippo pathway activity in LEC promotes their survival' is somewhat confusing as Yorkie is also part of the pathway and its activity is increased by downregulation of Hippo. Thus, the statement should be rephrased for better clarity, or the word 'pathway' could be removed.

- The nature of the tool used to downregulate ban - 'bantum-sponge'- should be described in the result section.

© 2026. Published by The Company of Biologists under the terms of the Creative Commons Attribution License (https://creativecommons.org/licenses/by/4.0/).

First revision

Author response to reviewers' comments

We thank the reviewers for their detailed, specific and constructive comments and respond to each comment below, with reference to improvements made to our manuscript in response to these critiques.

Reviewer #1

1. All figures where arrowheads are shown: The arrowheads should be enlarged and colored in against a nearly white background.

Author response: The size of arrowheads in all figures has been increased and white arrowheads have been recoloured to yellow to aid visualization where necessary.

2. Line 135-139: The authors showed that the defect in LEC apoptosis is caused by downregulation of the Hippo pathway. However, they did not investigate whether histoblast overgrowth (or proliferation?) is also caused by the Hippo pathway downregulation in this study. The authors should comment on this.

Author response: Previous work performed by Nicolas Tapon's Lab has explored a role for Hippo signalling in histoblast proliferation during abdominal morphogenesis. As work presented in a PhD thesis conducted in the Tapon Lab (Ainslie 2019) seemed to indicate that clonal downregulation of Hippo signalling components did not overtly affect histoblast nest growth, it was decided that this particular question would not be explored further in this study. As this PhD work has not been published, we felt it inappropriate to discuss these results in our manuscript.

3. Line 226: Correct (Fig. 5E) to (Fig. 5F and H). Line 226: (Fig. 5F) to (Fig. 5G). Line 227: (Fig. 5G) to (Fig. 5H).

Author response: Thank you for pointing this out. Corrections have since been made in the text.

4. Line 279-280: The authors should provide a brief explanation of 'bantam-sponge'.

Author response: An explanation of *bantam*-sponge has been added to lines 313 and 314 in the text.

Reviewer #2

1. Fig. 2A,C The authors state that LECs are completely cleared by 35h APF, however, there seems to be a substantial number of LEC nuclei still present at that time point (in particular at the dorsal midline). The images do not seem to represent the quantification for this time point. The authors should clarify.

Author response: While fluorescence is indeed still present along the length of the dorsal midline at 35h APF, this fluorescence is not believed to be attributable to intact LEC nuclei. After reaching the midline, LECs undergo apoptosis and fragments of intense fluorescent puncta associated with cellular debris linger along the midline for a short period of time. Higher magnification images are now provided in an updated version of Fig. 2 to make this distinction more apparent. As these fragments are unlikely to be representative of live LECs, they were excluded from LEC nuclei counts. It is not uncommon for a small number of intact LEC nuclei (<5) to still be present however, and this is reflected in the data. Additionally, this timing of 35h APF specifically refers to the A3 segment, with segments immediately posterior to the A3 segment experiencing slower LEC clearance and frequently presenting with considerable LEC nuclei at this time point. Clarification for this has also been provided in the text in line 157. Lastly, it should be noted that this timing of 35 h APF is not a novel assertion of this study, as a previous study has also reported complete clearance occurring in the A3

© 2026. Published by The Company of Biologists under the terms of the Creative Commons Attribution License (https://creativecommons.org/licenses/by/4.0/).

segment around this time point (Hoshika et al., 2020).

2. Fig. 2A,B The authors show images of the multiple segments of the dorsal abdomen, but analyze only segment A3. To increase clarity of the data, the authors should show (in addition) higher magnification views focusing on segment A3.

Author response: To aid in visualization of LEC nuclei used for analysis, higher magnification images focussing on the A3 segment have been added to Fig. 2A',B' and Fig. 4A',B'. A rolling ball background subtraction has been performed on these images in ImageJ to increase nuclei visibility.

3. Fig. 3B,S1 The authors claim that knockdown of wts using the Eip71CD-Gal4 driver results in LEC persistence. This conclusion has two limitations: First, Fig. 3B does not identify LECs, but shows brightfield images of the abdomen. Second, Fig. S1 does identify LECs, but only those expressing Diap1-GFP. We therefore cannot distinguish whether LECs no longer express the Diap1-GFP reporter or whether the LECs have been cleared. The number of cells that are not cleared might therefore be under-estimated. The authors should show fluorescence images of Eip71CD-Gal4 driving a nuclear marker and wts knockdown. Moreover, Fig. S1 is missing a negative control. The authors should therefore also show fluorescence images of Eip71CD- Gal4 driving a nuclear marker (without wts knockdown). Staging should preferably be h AEL (it is unclear what 'visible developmental features' are (legend to Fig. S1).

Author response: While not explicitly stated in the text, the choice to use *Diap1*-4.3-GFP to track LEC clearance in the presence of *Eip71CD*-GAL4 in lieu of a UAS-responsive fluorescent marker was a deliberate one, due to the strong expression of *Eip71CD* in the fat body. As the fat body directly underlies the developing pupal epithelium, additional fluorescence in the fat body obscures LEC nuclei and makes tracking their clearance difficult. This fat body expression of *Eip71CD* is unlikely to be involved in the resultant phenotype, as *pannier*-GAL4 shows no obvious fat body expression. An additional supplementary figure (Fig. S1) has been produced to track LEC clearance in control, *wts* RNAi, yki^{S168A} overexpression and *hpo* overexpression backgrounds and clarification stated in the text (lines 174 to 176). These images demonstrate that the adult phenotypes are due to persistent LECs, but also highlight the difficulties associated with using a UAS-responsive fluorescent protein to visualize LECs in the presence of this GAL4 driver. An attempt was made to image these pupae on a confocal microscope to separate out the fat body signal, but images were limited to a subset of LECs on the dorsal abdomen (containing the region contacting the coverslip) and the ventral abdomen was essentially unimageable. The strong expression of *Diap1*-4.3-GFP in LECs, with relatively little expression and Hippo-responsiveness in the fat body, circumvents the issues displayed in this figure.

In relation to concerns around underestimation of LEC clearance using *Diap1*-4.3-GFP due to a loss of *Diap1* expression, an experiment was performed viewing LEC clearance in a *wts* RNAi pupae expressing both *Diap1*-4.3-GFP and UAS-His2A-RFP simultaneously (Reviewer figure 1). From this figure it can be seen that there is strong overlap between *Diap1*-4.3-GFP fluorescence and His2A-RFP in persistent LECs at ~96 h APF, with no obvious signs of cells losing *Diap1*-4.3-GFP expression. The only clear instance where these two fluorescent proteins do not overlap is in condensed nuclei, which appear to lack *Diap1*-4.3-GFP fluorescence.

To address the other points in this comment relating to a lack of a negative control and unclear developmental timings, control and *wts* RNAi pupae expressing *Diap1*-4.3-GFP have been reimaged with strict timings and an updated Fig. S2 (Previously Fig. S1) has been included.

4. Fig. S2 is missing a negative control. The authors should show fluorescence images of Eip71CD-Gal4 driving a nuclear marker (without Yki-S168A).

Author response: Similar to the previous comment, pupae overexpressing yki^{S168A} under *Eip71CD*-GAL4 have been imaged with a UAS-responsive fluorescent marker and included in Fig. S1C,C'. An updated Fig. S3 (Previously Fig. S2) has also been produced displaying LEC clearance in the presence of *Diap1*-4.3-GFP with an appropriate control and strict developmental timings.

5. Fig. S3, line 175 The authors claim that pupae exhibited gaps in the developing epithelium.

© 2026. Published by The Company of Biologists under the terms of the Creative Commons Attribution License (https://creativecommons.org/licenses/by/4.0/).

These gaps are difficult to see. The authors should show more appropriate images.

Author response: We agree with the reviewer's assessment. While the spreading of histoblasts appears disrupted, the images do not provide strong evidence of a gap in the epithelium. Using *Diap1*-4.3-GFP to track LEC clearance in the presence of *hpo* overexpression is difficult because the reporter is Hippo-responsive. To address this, an additional experiment was performed using E-Cad-GFP to visualize adherens junctions in both LECs and histoblasts in the presence/absence of *hpo* overexpression under *Eip71CD*-GAL4. Reimaging of pupae expressing *Diap1*-4.3-GFP was first performed to determine the time frame in which these gaps occur, and these results are displayed in Fig. S4. Images of pupae at 32h APF expressing E-Cad-GFP in the presence or absence of *hpo* overexpression are included in Fig. S5, providing clearer representation of epithelial disruptions.

6. Fig. 4B, line 191 The authors claim signs of condensed nuclei and apoptotic cells at 15 h APF in sd knockdowns. The authors should explain how they identify condensed nuclei and apoptotic cells and show higher magnification views to corroborate their findings. Moreover, controls apparently also show condensed nuclei at this time point. The authors should quantify the number of condensed nuclei in both genotypes.

Author response: Condensed nuclei were identified as small and intense points of fluorescence present in abdominal segments. Typically, these nuclei only become apparent in appreciable numbers during the later stages of abdominal development, and as such they were assumed to be representative of apoptotic cells. To better visualize these condensed nuclei and to confirm they are indeed representative of apoptotic cells, an additional experiment was performed using confocal microscopy and the caspase reporter GC3Ai. The results of this experiment are depicted in Fig. 4D-G, with higher magnification views illustrating the disrupted nuclear architecture of these condensed nuclei. Confirming these cells are apoptotic, strong GC3Ai fluorescence was found to be present in cells which displayed nuclear condensation. These results also highlight the propensity of *sd* RNAi LECs to activate caspases and progress through apoptosis earlier than control pupae. An explanation of these results has been added to lines 212 to 226.

7. Fig. 5C Knockdown of sd at 25C results in decreased DIAP1 expression, but does not influence LEC removal. Does this mean that DIAP1 expression is not important for LEC removal, which appears to be inconsistent with Kester and Nambu, 2011? The authors should clarify.

Author response: The simplest explanation for the seemingly normal clearance of *sd* RNAi LECs at 25°C is that, due to inefficient knockdown, a functional threshold has not been passed; expression of *Diap1* (and other Hippo targets) is reduced but not by enough to perturb LEC clearance. Increasing the efficiency of *sd* RNAi at 29°C is sufficient to cause premature LEC apoptosis (Fig S6C,D), presumably because the threshold is now breached. Our results at 25°C as such do not necessarily assert that *Diap1* is not important for LEC removal but rather imply that a certain level of *Diap1* expression is required for continued LEC survival. It should also be noted that while Kester and Nambu 2011 did indeed show that LECs are sensitive to changes in *Diap1* expression, they did so through overexpression experiments and did not examine any role of endogenously expressed *Diap1* during LEC development. While it seems highly likely that endogenous *Diap1* expression plays a role in regulating normal LEC clearance, to our knowledge no such study has been produced explicitly demonstrating this function during development.

8. Fig. 7A' The authors claim that, when DIAP1 is overexpressed, surviving LECs persist underneath the adult epithelium. This is difficult to see from the shown image. The authors should show better images.

Author response: The notion that *Diap1* overexpression LECs persist beneath the epithelium is not an assertion of this study, but rather a result presented in Ninov et al., 2007 (This effect was actually attributed to p35 overexpression rather than *Diap1*). As such, no images have been produced in this study indicating that this is the case. The wording of this statement has been adjusted to make clear that this is an assertion of a previous study instead of our own.

© 2026. Published by The Company of Biologists under the terms of the Creative Commons Attribution License (https://creativecommons.org/licenses/by/4.0/).

9. Line 257 The authors claim that LECs persisting following wts knockdown do not extrude basally. The authors should indicate to which figure they refer to.

Author response: Our claim that *wts* RNAi LECs do not basally extrude is an assumption based on our observation that these persistent LECs protrude apically above the adult cuticle (See Fig. 1B'). We do not have any experimental data showing that the protrusion of these cells is related to defective basal extrusion. Language in the text has been changed to reflect this.

10. Fig. 7F The authors link clumps of tissue at the posterior most end of flies induced by DIAP1 and ban co-expression with consequence of wts knockdown (Fig. 3B). This resemblance is unclear and the authors should clarify.

Author response: A link between the phenotype in Fig. 7F and the LEC persistence phenotype produced by *wts* RNAi was proposed due to the characteristic spherical nature of persistent LECs visible in these clumps. To confirm that this protrusion from the posterior abdomen is indeed associated with LEC persistence, the experiment was repeated with the addition of a UAS-responsive His2A-RFP as a marker of LEC nuclei. The results of this experiment have been included in Fig. 7F',F'', with LEC nuclei readily visible in these clumps of tissue.

11. Fig. S7A The authors claim that co-expression of bantam-sponge suppresses the abdominal phenotype induced by DIAP1 overexpression. However, expression of two UAS-genes from a single pannier-Gal4 driver may sequester Gal4 and hence decrease DIAP1 expression. The authors should co-express UAS-lacZ or similar with DIAP1 and test whether the DIAP1-induced abdominal phenotype is present (or suppressed).

Author response: Thank you for pointing this out. An additional cross has been performed using a UAS-responsive RNAi line targeting GFP. As seen in Fig. S9D, the addition of a second UAS element alone does not appear to be sufficient to rescue the abdominal phenotype produced by *Diap1* overexpression.

12. Fig. 1. The authors should use A,B,C to label individual images and describe the images in the legend. As it stands, the two images on the right are not mentioned in the legend.

Author response: This figure has been updated to include lettering as labels for each individual image, and additional explanation has been provided in the figure caption.

13. Fig. S3 This figure appears to be insufficiently described in the results part, e.g. the data in panels C and D are not mentioned.

Author response: An additional sentence has been added to the text (Lines 190 to 192) to include a description of the result shown in Fig. S5B (Previously Fig. S3D).

14. Fig. 5F-H, line 226 The panels in the text relating to Yki subcellular distribution should read F,G,H, not E,F,G

Author response: Thank you for informing us of this error. References to the appropriate figure panels have since been added in the text.

Reviewer #3

The manuscript by Lyon et al. investigates the role of Hippo signaling in regulating the survival of abdominal larval epithelial cells (LECs) during pupal metamorphosis. The central finding is that elevated Hippo pathway activity, resulting in reduced nuclear Yorkie, promotes LEC elimination. The study includes systematic manipulation of multiple Hippo pathway components, however, the primary observations are largely consistent with the well-established role of Hippo signaling in controlling cell survival. Previous studies have already demonstrated that overexpression of Diap1 enhances LEC survival, making the general conclusion that Hippo activation promotes cell elimination somewhat expected.

Authors response: While the Hippo pathway certainly has been well-documented to affect cell

© 2026. Published by The Company of Biologists under the terms of the Creative Commons Attribution License (https://creativecommons.org/licenses/by/4.0/).

survival, instances of this occurring in a manner dependent upon endogenous Sd-mediated transcription are relatively scant within the literature. Furthermore, while papers such as Kester and Nambu 2011 have shown that overexpressing *Diap1* can influence LEC survival, no study has been performed examining the role of endogenous *Diap1* in regulating LEC clearance. Our findings on the role of endogenous *Diap1* expression in LEC survival and the association of this function with temporal changes in Hippo pathway activity thus provide novel insights not previously reported.

1. First, the authors should clarify whether these intensity differences are inherent to the genotypes or due to differences in imaging parameters. Second, within the same sample/segment, there appear to be variations in RFP intensity, which could introduce inaccuracies in automated or threshold-based cell counting.

Author response: The reviewer is correct that the intensities of RFP fluorescence in LEC nuclei vary between genotypes, with *wts* RNAi nuclei appearing brighter than controls, while *sd* RNAi nuclei are seemingly dimmer. All pupae were imaged using the same microscope settings, and as such differences in RFP intensities between genotypes are believed to be inherent to the genotypes themselves. These differences may be due to changes in endoreplication rates experienced by these LECs during development, with *wts* RNAi LECs having a higher ploidy while *sd* RNAi LECs are lower. The *pannier* GAL4 driver is expressed in LECs throughout larval development when the cells are performing endoreplication, and *ban* has previously been shown to influence endoreplication rates in LECs (Jiang et al., 2014). These potential differences in endoreplication are, however, unlikely to be a factor influencing LEC clearance. For instance, previous work performed in our lab has found that upregulating insulin signalling greatly increased LEC ploidy but did not disrupt LEC clearance. Additionally, endoreplication is even less likely to be a factor influencing LEC clearance under the *Eip71CD* GAL4 driver, as expression of this driver only begins during the late third instar larval stage, when LEC growth has ceased. As to the second point, there does indeed appear to be some level of variation in His2A-RFP levels between nuclei within the same abdominal segment. As *pannier* functions as a segmentation gene, it is speculated this effect may be due to variation in *pannier*-GAL4 occurring within segments due to a patterning effect of *pannier* expression. Concerns around the effect this may have on automated or threshold-based cell counting have hopefully been circumvented through manual counting.

2. Importantly, the manuscript lacks methodological details describing how LEC numbers were quantified. Given that these data form the basis for temporal conclusions, the quantification method must be described in sufficient detail.

Author response: Additional detail surrounding image analysis has been provided in the methods section. For the quantification of LEC nuclei numbers, manual analysis was chosen to eliminate issues associated with background fluorescence which became apparent when thresholding nuclei in ImageJ.

3. To test cell survival/cell death authors should consider taking direct approach. The Apoliner assay driven by pnr-Gal4 at one time-point is not sufficient on its own to support the conclusions. Cell death should be tested at multiple time points in relevant genotypes, possibly adding Dcp1 staining to avoid potential issues with Apoliner expression levels (RFP in 6A appears differently localized compared with 6B, making it difficult to judge the Apoliner levels).

Author response: We agree that a single time point is insufficient to determine that caspase activation is disrupted in LECs by *wts* RNAi. To rectify this, a second, later time point has been imaged, viewing changes in Apoliner cleavage at 30h APF. The results of this experiment have been included in Fig. 6C,D,E, and indicate that Apoliner cleavage remains significantly reduced relative to controls even during the later stages of LEC clearance. In terms of concerns around differences in Apoliner expression levels between genotypes, while we appreciate the suggestion of using Dcp1 staining as a direct measure of caspase activity, such an experiment would require the imaging of fixed samples. Dissecting and fixing pupae could inadvertently induce changes in caspase activation and there were concerns about how that would complicate interpretation relative to a live caspase sensor. As an alternative to Dcp1 staining, live pupae were instead imaged with a second caspase sensor, GC3Ai. This sensor consists of a non-fluorescent GFP protein which refolds to become fluorescent upon cleavage by effector caspases. As can be seen

© 2026. Published by The Company of Biologists under the terms of the Creative Commons Attribution License (https://creativecommons.org/licenses/by/4.0/).

in Reviewer figure 2, patterns of GC3Ai fluorescence in control and *wts* RNAi LECs at both 26h and 30h APF closely mirror the results obtained using Apoliner.

4. A more detailed analysis distinguishing isolated versus clustered cell death, similar to the approach used in Yuswan et al., 2024, would be helpful in determining whether the temporal effects of Hippo signaling preferentially influence a specific mode of LEC elimination.

Author response: The question of whether Hippo signalling preferentially influences isolated or clustered cell death is an intriguing one that we have not considered closely. We would be keen to pursue this line of enquiry in the future but given the extent of Yuswan and colleague's work we feel such a study would constitute a follow-up manuscript.

5. Since, not all LECs die at the same time, I was wondering if the nuclear localization or exclusion of Yki correlates with the cell death. Testing this directly would help clarify the relationship between Yki activity and LEC elimination.

Author response: We appreciate this suggestion and agree that it would be interesting to correlate the time of LEC apoptosis with changes in Yki localisation on a cell by cell basis. However, this would require extensive live imaging experiments and cell tracking which we feel is beyond the scope of the current study, and as such we have instead proceeded with a population-wide analysis of Yki localisation and apoptosis at discrete time points.

6. Yki is shown to be excluded from the nucleus until 14h APF. While authors have discussed plausible mechanisms this temporal regulation, they should also address why cell death levels remain low during the 0-13 hrs APF period, despite apparently high Hippo activity.

Author response: Our current explanation for a lack of apoptosis during this period is that there is an absence of apoptotic stimuli. LECs may not be receiving any signals to undergo apoptosis at this time, and thus a lack of anti-apoptotic factors associated with high levels of Hippo signalling does not result in apoptosis. This notion is supported by levels of *Diap1*-4.3-GFP expression being very low in LECs during this period (<14 h APF), in addition to being low throughout the entirety of larval development. Under this theory, the translocation of Yki into the nucleus shortly after head eversion either coincides with or pre-empts the induction of an apoptotic stimulus, with elevated expression of anti-apoptotic factors acting to keep LECs alive during the period before histoblasts are ready to form the adult epithelium. Where this apoptotic stimulus originates from is still an unanswered question. It may arise in response to histoblast proliferation, or potentially other factors such as loss of basement membrane contacts through ECM and larval muscle degradation. Follow-up studies will be required to provide such an answer.

7. Abstract and elsewhere: Statement: "Downregulating Hippo pathway activity in LEC promotes their survival' is somewhat confusing as Yorkie is also part of the pathway and its activity is increased by downregulation of Hippo. Thus, the statement should be rephrased for better clarity, or the word 'pathway' could be removed.

Author response: While we understand that the wording of statements such as these comes across as confusing, they do follow normal conventions established in the literature. Typically, statements discussing changes in Hippo pathway activity are presumed to relate to the activity of upstream components of the pathway such as Hpo and Wts, with these changes in turn expected to have an inverse effect on Yki activity.

8. The nature of the tool used to downregulate ban - 'bantum-sponge'- should be described in the result section.

Author response: A description of *bantam*-sponge has been added to the text in lines 313 and 314.

Reviewer figures:

© 2026. Published by The Company of Biologists under the terms of the Creative Commons Attribution License (https://creativecommons.org/licenses/by/4.0/).

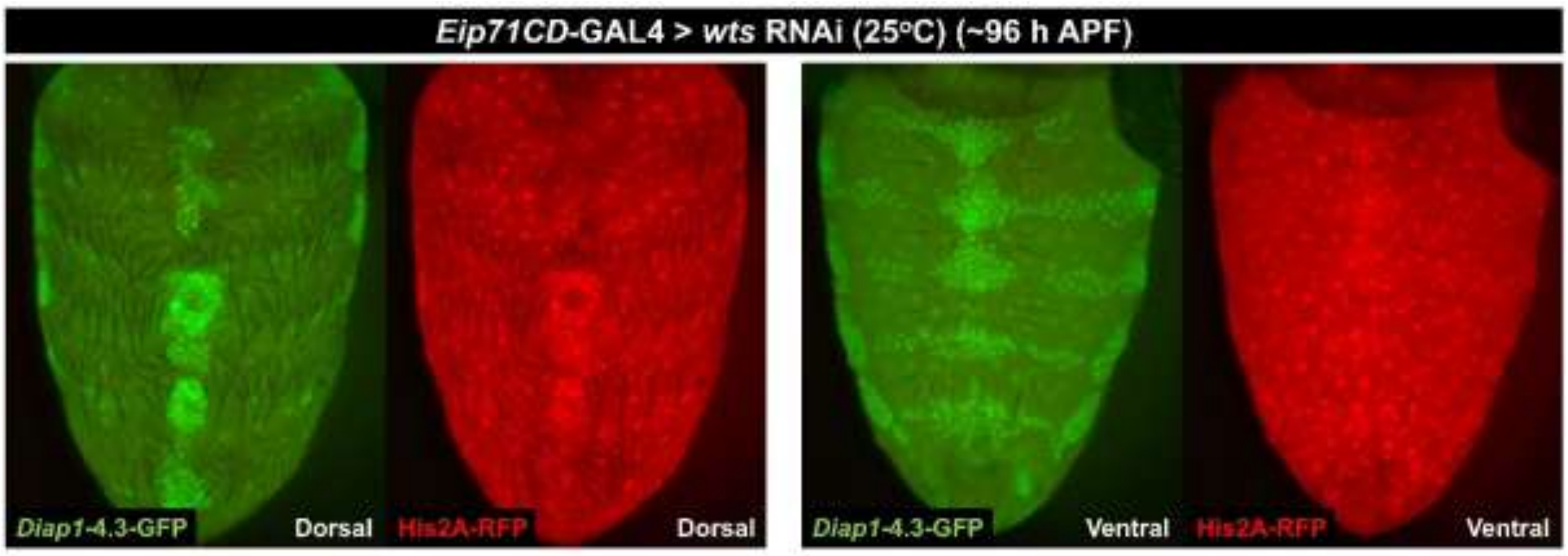

Reviewer figure 1. *wts* RNAi pupae simultaneously expressing *Diap1*-4.3-GFP and UAS-His2A- RFP under *Eip71CD*-GAL4. In the dorsal and ventral abdomen, close correlation is seen between His2A-RFP driven by the GAL4 line and the transcriptional reporter *Diap1*-4.3-GFP in persistent LECs, indicating that *Diap1*-GFP expression is maintained in these cells into adulthood. His2A-RFP driven by *Eip71CD*-GAL4 expression in the fat bodies (which expresses *Diap1*-4.3-GFP to a weaker extent than LECs and has little Hippo pathway responsiveness) obscures visualization of persistent LECs throughout the abdomen.

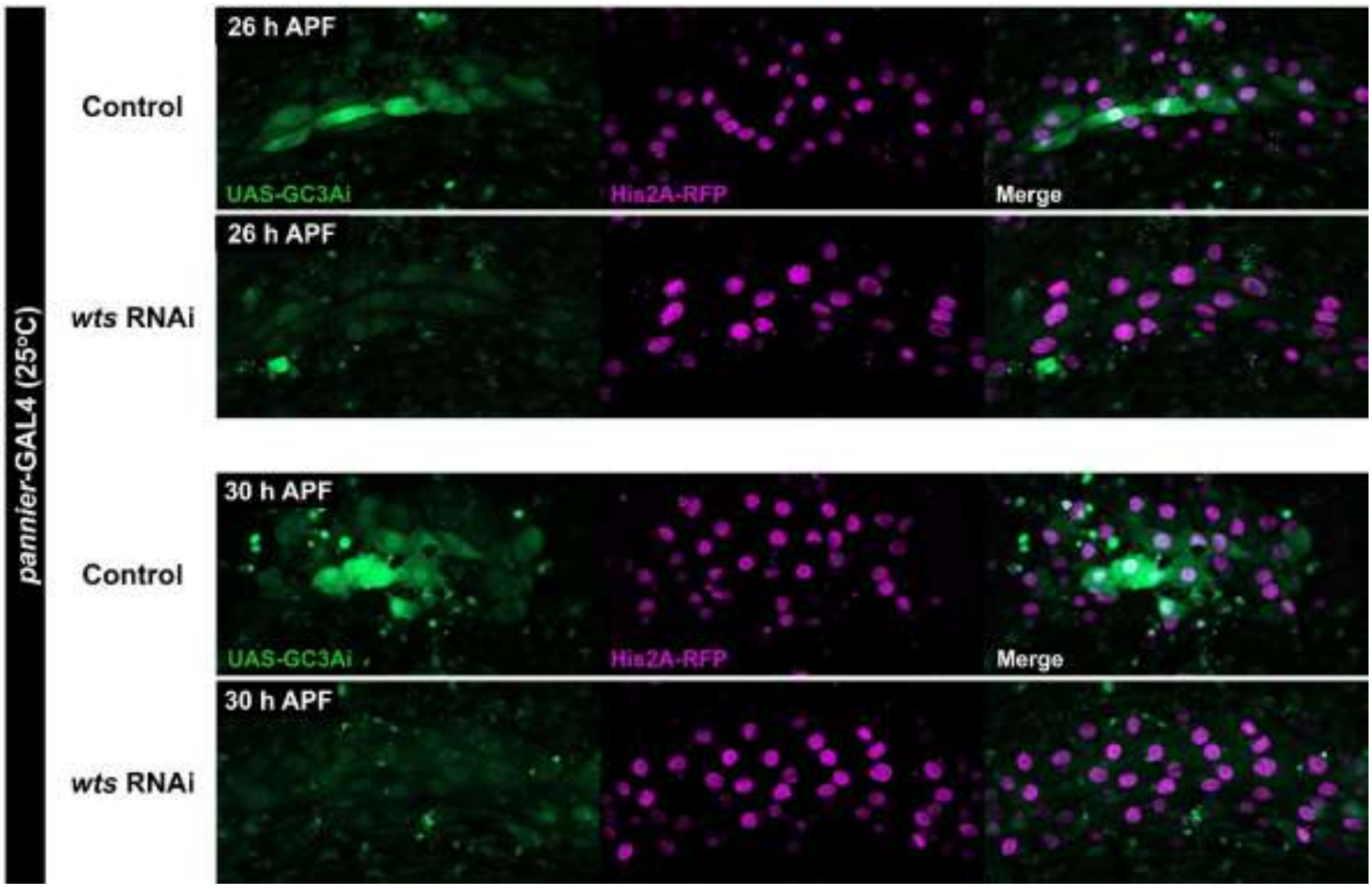

Reviewer figure 2. The caspase reporter GC3Ai indicates that caspase activation is reduced in *wts* RNAi LECs at both 26 h and 30 h APF relative to controls. This provides an example of reduced caspase activation associated with *wts* RNAi using a second caspase sensor, in addition to Apoliner, adding strength to our argument that *wts* RNAi significantly impedes LEC apoptosis.

Second decision letter

© 2026. Published by The Company of Biologists under the terms of the Creative Commons Attribution License (https://creativecommons.org/licenses/by/4.0/).

MS ID#: jcs.264798R1

MS TITLE: The Hippo signalling pathway regulates the timing of epithelial cell replacement during *Drosophila* abdominal morphogenesis

AUTHORS: Kyle Lyon; Samuel A. Manning; Richard Burke

ARTICLE TYPE: Research Article

Dear Dr Burke,

We have now reached a decision on the above manuscript.

As you will see, the reviewers gave favourable reports but raised some points that will require amendments or explanations to your manuscript. I hope you will be able to provide these, as I would like to accept your paper.

Reviewer 1

Lyon et al. have addressed my comments appropriately. The revised manuscript adequately addresses my concerns, and I believe it is suitable for publication.

Reviewer 2

SUMMARY OF THE ADVANCE MADE IN THIS PAPER AND ITS POTENTIAL SIGNIFICANCE TO THE FIELD

SUGGESTIONS TO AUTHORS

The authors have carefully and satisfactorily addressed all my comments.

Reviewer 3

SUGGESTIONS TO AUTHORS

Minor comments
I still have a minor technical concern regarding the use of the GC3Ai caspase sensor instead of the Dcp1 staining. Since GC3Ai is also UAS-driven and the control used in these experiments is w1118 rather than a genotype carrying UAS-lacZ (or similar neutral UAS), the number of UAS transgenes is not balanced between the experiments and controls. This is not a major concern for Figure 4D-G, as sd depletion results in a clear increase in GC3Ai signal, which is unlikely to be due to Gal4 titration. However, in the wts RNAi experiments both GC3Ai and Apoliner signals are reduced (although the genotype of the control is not explicitly mentioned for these experiments, I assume it is w1118, as indicated elsewhere in the manuscript). While I appreciate that the marked difference between 26 and 30 h APF in the control, compared with the wts RNAi genotype, suggesting that wts depletion has an effect, the conclusion would nevertheless be more convincing if the UAS copy number were balanced between the genotypes for at least one of the two reporters.

Second revision

Author response to reviewers' comments

Reviewer #3

1. I still have a minor technical concern regarding the use of the GC3Ai caspase sensor instead of the Dcp1 staining. Since GC3Ai is also UAS-driven and the control used in these experiments is w1118 rather than a genotype carrying UAS-lacZ (or similar neutral UAS), the number of UAS transgenes is not balanced between the experiments and controls. This is not

© 2026. Published by The Company of Biologists under the terms of the Creative Commons Attribution License (https://creativecommons.org/licenses/by/4.0/).

a major concern for Figure 4D-G, as sd depletion results in a clear increase in GC3Ai signal, which is unlikely to be due to Gal4 titration. However, in the wts RNAi experiments both GC3Ai and Apoliner signals are reduced (although the genotype of the control is not explicitly mentioned for these experiments, I assume it is w1118, as indicated elsewhere in the manuscript). While I appreciate that the marked difference between 26 and 30 h APF in the control, compared with the wts RNAi genotype, suggesting that wts depletion has an effect, the conclusion would nevertheless be more convincing if the UAS copy number were balanced between the genotypes for at least one of the two reporters.

Author response: The reviewer is correct that the control background used in both the Apoliner and GC3Ai experiments was w^{1118}, with there being an imbalance in the number of UAS elements present in the control relative to the experimental genotype. While unlikely, we agree that this leaves the possibility that the effect of *wts* RNAi on both Apoliner and GC3Ai is reflective of a change in GAL4 titration rather than an effect on caspase activation. To address this concern, an additional cross was performed to examine the effect of a UAS-responsive *LacZ* RNAi line on Apoliner-GFP localization across pupal development. From the results of this cross (Reviewer figure 1), it can be seen that there is no significant difference between Apoliner GFP localization in the presence of *LacZ* RNAi relative to a w^{1118} background at both 26 h APF and 30 h APF. As such, we can be confident the effect of *wts* RNAi on Apoliner is not simply associated with the presence of an additional UAS element relative to the control, instead being reflective of a bona fide change in caspase activation. The Apoliner GFP data in Figure 6 has been updated accordingly to present a comparison between *LacZ* RNAi and *wts* RNAi.

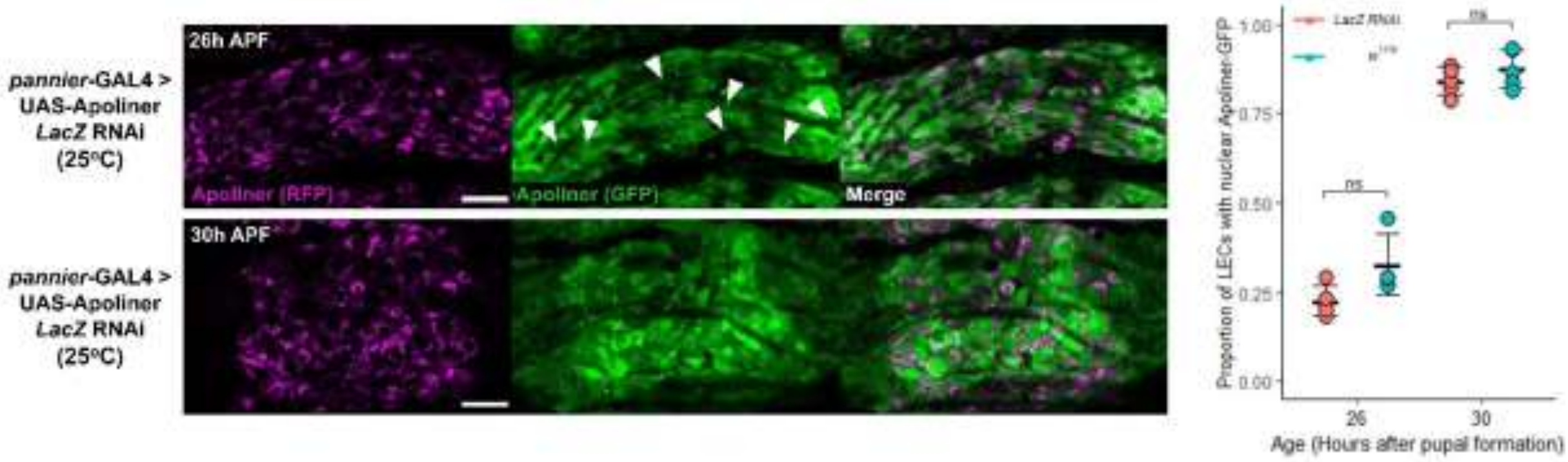

Reviewer figure 1. *LacZ* RNAi has no obvious effect on Apoliner-GFP localization across pupal development relative to a w^{1118} background. The caspase sensor Apoliner indicates cells experiencing caspase activation through the presence of nuclear Apoliner-GFP localization. At both 26 h and 30 h APF, it can be seen that there is no significant difference in the proportion of cells displaying nuclear Apoliner-GFP in a *LacZ* RNAi background relative to a w^{1118} control.

<u>Third decision letter</u>

MS ID#: jcs.264798R2

MS Title: The Hippo signalling pathway regulates the timing of epithelial cell replacement during *Drosophila* abdominal morphogenesis

Authors: Kyle Lyon; Samuel A. Manning; Richard Burke

Article Type: Research Article

Dear Dr Burke,

© 2026. Published by The Company of Biologists under the terms of the Creative Commons Attribution License (https://creativecommons.org/licenses/by/4.0/).

I am happy to tell you that your manuscript has been accepted for publication in Journal of Cell Science, pending standard publication integrity checks.

© 2026. Published by The Company of Biologists under the terms of the Creative Commons Attribution License (https://creativecommons.org/licenses/by/4.0/).
